# Exercise and Psychosexual Education to Improve Sexual Function in Men With Prostate Cancer: A Randomized Clinical Trial

**DOI:** 10.1001/jamanetworkopen.2025.0413

**Published:** 2025-03-12

**Authors:** Daniel A. Galvão, Robert U. Newton, Dennis R. Taaffe, Prue Cormie, Oliver Schumacher, Christian J. Nelson, Robert A. Gardiner, Nigel Spry, David Joseph, Colin Tang, Hao Luo, Raphael Chee, Dickon Hayne, Suzanne K. Chambers

**Affiliations:** 1 Exercise Medicine Research Institute, Edith Cowan University, Perth, Australia; 2 School of Medical and Health Sciences, Edith Cowan University, Perth, Australia; 3 Peter MacCallum Cancer Centre, Melbourne, Australia; 4 Sir Peter MacCallum Department of Oncology, The University of Melbourne, Melbourne, Australia; 5 Department of Psychiatry and Behavioral Sciences, Memorial Sloan Kettering Cancer Center, New York, New York; 6 Centre for Clinical Research, University of Queensland, Brisbane, Australia; 7 Department of Urology, Royal Brisbane and Women’s Hospital, Brisbane, Australia; 8 Department of Radiation Oncology, Sir Charles Gairdner Hospital, Perth, Australia; 9 UWA Medical School, University of Western Australia, Perth, Australia; 10 Radiation Oncology, Genesis Care, Perth, Australia; 11 Department of Urology, Fiona Stanley Hospital, Murdoch, Western Australia, Australia; 12 Faculty of Health Sciences, Australian Catholic University, Brisbane, Australia

## Abstract

**Importance:**

Sexual dysfunction is a common adverse effect of prostate cancer treatment, and current management strategies do not adequately address physical and psychological causes. Exercise is a potential therapy in the management of sexual dysfunction.

**Objective:**

To investigate the effects of supervised, clinic-based, resistance and aerobic exercise with and without a brief psychosexual education and self-management intervention (PESM) on sexual function in men with prostate cancer compared with usual care.

**Design, Setting, and Participants:**

A 3-arm, parallel-group, single-center randomized clinical trial was undertaken at university-affiliated exercise clinics between July 24, 2014, and August 22, 2019. Eligible participants were men with prostate cancer who had previously undergone or were currently undergoing treatment and were concerned about sexual dysfunction. Data analysis was undertaken October 8 to December 23, 2024.

**Interventions:**

Participants were randomized to (1) 6 months of supervised, group-based resistance and aerobic exercise (n = 39 [34.8%]), (2) the same exercise program plus PESM (n = 36 [32.1%]), or (3) usual care (n = 37 [33.0%]). Exercise was to be undertaken 3 days per week.

**Main Outcomes and Measures:**

The primary outcome was sexual function assessed with the International Index of Erectile Function (IIEF). Secondary outcomes included body composition, physical function, and muscle strength. Analyses were undertaken using an intention-to-treat approach.

**Results:**

In total, 112 participants (mean [SD] age, 66.3 [7.1] years) were randomized. Mean adjusted difference in IIEF score at 6 months favored exercise compared with usual care (3.5; 95% CI, 0.3-6.6; *P* = .04). The mean adjusted difference for intercourse satisfaction was not significant (1.7; 95% CI, 0.1-3.2; *P* = .05). PESM did not result in additional improvements. Compared with usual care, exercise also significantly improved fat mass (mean adjusted difference, −0.9 kg; 95% CI, −1.8 to −0.1 kg; *P* = .02), chair rise performance (mean adjusted difference, −1.8 seconds; 95% CI, −3.2 to −0.5 seconds; *P* = .002), and upper (mean adjusted difference, 9.4 kg; 95% CI, 6.9-11.9 kg; *P* < .001) and lower (mean adjusted difference, 17.9 kg; 95% CI, 7.6-28.2 kg; *P* < .001) body muscle strength.

**Conclusions and Relevance:**

In this randomized clinical trial of supervised exercise, erectile function in patients with prostate cancer was improved. PESM resulted in no additional improvements. Patients with prostate cancer should be offered exercise following treatment as a potential rehabilitation measure.

**Trial Registration:**

ANZCTR Identifier: ACTRN12613001179729

## Key Points

**Question:**

Does 6 months of supervised resistance and aerobic exercise improve sexual function in men with prostate cancer, and are positive effects enhanced when combined with a brief psychosexual self-management intervention?

**Findings:**

In this randomized clinical trial including 112 patients with prostate cancer, exercise improved erectile function compared with usual care. Brief psychosexual education with self-management did not result in additional improvements.

**Meaning:**

Results of this study suggest that for men with prostate cancer concerned about sexual dysfunction, exercise is an effective intervention to improve sexual function and should be considered as an integral part of treatment.

## Teaser

This randomized clinical trial reports the efficacy of a supervised exercise intervention for sexual function in men with prostate cancer and whether addition of a brief psychosexual education and self-management intervention results in more pronounced effects compared with supervised exercise alone.

## Introduction

Sexual function is adversely affected following prostate cancer treatment.^1^ The decline in erectile function (the most common factor impacting sexual function)^2^ is progressive even 15 years after prostatectomy and radiotherapy (although age is a potential contributing factor),^3^ with other aspects such as sexual desire, altered ejaculatory and/or orgasmic function, and modifications in partner relationships also contributing to sexual dysfunction.^1,2^ Current management of sexual dysfunction in men with prostate cancer predominantly involves pharmacological intervention to address the direct physiological effects of prostate cancer treatment on erectile function.^4^ However, sexual dysfunction is complex and there are physical, psychological, and relationship effects of prostate cancer treatment that contribute to such impairment.^1^ Importantly, most men report that they are not offered helpful interventions to support sexual function after prostate cancer treatments.^5^

Exercise is a potential therapy in the management of sexual function for men with prostate cancer as it can counteract physical (eg, body feminization, loss of muscle mass and strength, and declining physical function as a result of androgen deprivation therapy [ADT])^6^ and psychological^7^ adverse effects of treatment implicated with sexual dysfunction.^8^ Exercise can also promote improved feelings of masculinity^9^ and preserve libido.^10^ Further, multimodal psychosocial and psychosexual interventions have been shown as acceptable to men with prostate cancer and to improve mental health outcomes and quality of life, as well as increase sexual satisfaction and decrease sexual bother.^11^ However, there is limited research on the effects of exercise and the potential combination of exercise and psychosexual education for sexual function in men with prostate cancer.

Herein we report the efficacy of a supervised exercise intervention on sexual function in men with prostate cancer concerned about sexual dysfunction and whether exercise combined with a brief psychosexual education and self-management intervention (PESM) results in more pronounced effects on sexual function compared with supervised exercise alone. Changes in sexual function assessed by the International Index of Erectile Function (IIEF) over 6 months served as the primary study end point. Secondary outcomes included physical factors (ie, body composition, functional capacity, and muscle strength) associated with sexual dysfunction. We hypothesized that exercise would improve sexual function in men with prostate cancer concerned about sexual dysfunction compared with standard medical care. Moreover, we hypothesized that exercise combined with PESM would result in improvements in sexual function that exceed those observed with exercise alone.

## Methods

### Study Design, Participants, and Procedures

**Figure 1.**
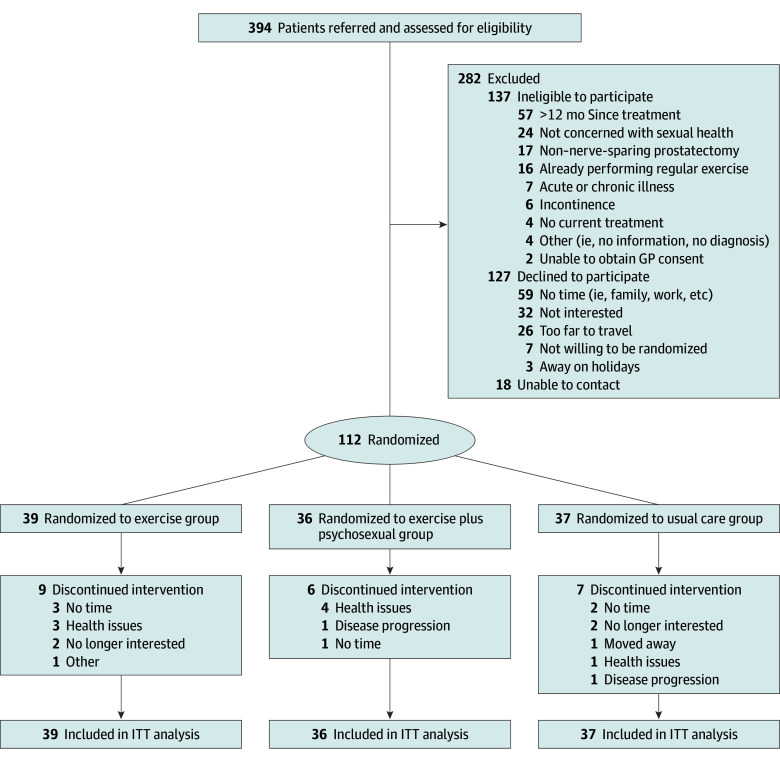
CONSORT Diagram GP indicates general practitioner; ITT, intention to treat.

This was a 3-arm, single-blinded (investigators blinded), parallel-group, single-center randomized clinical trial. The final trial protocol and statistical analysis plan are included in Supplement 1, and the study adhered to the Consolidated Standards of Reporting Trials (CONSORT) reporting guideline. Patients with prostate cancer were recruited in Perth, Australia, between July 24, 2014, and December 20, 2018, by invitation from their urologist or oncologist and referred to the study coordinator for eligibility screening. Three hundred and ninety-four men were referred and screened. Their progress through the study is shown in Figure 1. Inclusion criteria were: (1) concern about sexual function as assessed by an IIEF overall satisfaction score of less than 8, indicating moderately to very dissatisfied (scores range from 2-10)^12^ and/or an Expanded Prostate Cancer Index Composite (EPIC) sexual bother score of greater than 8 (ie, a small to big problem) indicating symptomatic dysfunction (scores range from 1-17, calculated by summing raw scores)^13^; (2) prior or current treatment for prostate cancer, including prostatectomy, radiotherapy, or ADT; and (3) physician consent. Exclusion criteria consisted of (1) non–nerve-sparing prostatectomy; (2) more than 12 months since prostatectomy or completion of radiotherapy or ADT (initially >6 months and amended to facilitate recruitment); (3) incontinence defined as requiring the use more than 1 pad in a 24-hour period; (4) already performing regular exercise defined as undertaking structured aerobic or resistance training at least 2 times per week within the past 3 months; (5) acute illness or any musculoskeletal, cardiovascular, and/or neurological disorder that could inhibit exercise or put participants at risk from exercising; and (6) inability to read and speak English. Following a familiarization session and baseline assessments, participants were randomized to an exercise group, exercise and PESM group, or usual care group in a 1:1:1 ratio. Participants were stratified by (1) age (<60 or ≥60 years), (2) current sexual activity (yes or no) as assessed by the sexual activity score in the prostate cancer module of the European Organisation for Research and Treatment of Cancer (EORTC) quality of life questionnaire (QLQ-PR25),^14^ (3) previous prostatectomy (yes or no), (4) previous radiotherapy (yes or no), and (5) previous or current ADT (yes or no). Randomization was performed independently by the National Health and Medical Research Council Clinical Trials Center, Sydney, Australia. The study was approved by the Human Research Ethics Committee at Edith Cowan University and associated hospitals in Perth, Australia, and all participants provided written informed consent. The detailed methods of the study protocol have been published elsewhere.^15^

### Interventions

Exercise consisted of aerobic and resistance training undertaken 3 days per week for 6 months. All exercise sessions were supervised by an accredited exercise physiologist and conducted in small groups of as many as 10 to 12 participants at various university-affiliated exercise clinics in Perth. The aerobic component of the program involved 20 to 30 minutes of cardiovascular exercise performed at moderate to vigorous intensity (approximately 60%-85% of estimated maximal heart rate) on a treadmill, cycling or rowing ergometer, or elliptical or cross trainer. In addition, participants were encouraged to undertake further home-based aerobic exercise and accumulate a total of at least 150 minutes of moderate-intensity aerobic exercise per week. Resistance training consisted of 6 to 8 exercises targeting the major upper and lower body muscle groups with intensity ranging from 6 to 12 repetitions maximum using 1 to 4 sets per exercise. The exercise program was progressive in nature and periodized, altering emphasis on exercise intensity and volume. Sessions commenced with a 10-minute warm-up consisting of low-intensity aerobic exercise and stretching and concluded with a 5-minute cool-down consisting of stretching.

Participants in the exercise plus PESM group completed the same exercise intervention described above as well as a brief intervention that addressed psychological and sexual well-being. A low-intensity psychological care approach was used to maximize uptake and facilitate translation.^16^ At baseline, participants attended a brief face-to-face PESM session with their exercise physiologist, who received training in how to deliver the intervention. Session content included stress management, problem-solving coping for treatment challenges, and goal setting for sexual rehabilitation. The intervention used a cognitive behavioral and adult learning approach where men self-selected rehabilitation goals.^17^ To support self-management, participants received a published self-help book for men with prostate cancer and their partners,^18^ a study-specific tip sheet about treatments for erectile dysfunction and goal setting for sexual rehabilitation, a progress journal, and audio resources for stress management. Participants in the usual care group received standard medical care and were asked to maintain their current physical activity level for 6 months.

### Outcome Measures

The primary outcome was sexual function across multiple domains assessed at baseline and 6 months using the IIEF-15 (erectile function, orgasmic function, sexual desire, intercourse satisfaction, and overall satisfaction), EPIC (sexual function), and EORTC QLQ-PR25 (sexual activity). Secondary outcomes were body composition, physical function, and muscle strength. Lean mass and fat mass were assessed by dual-energy x-ray absorptiometry (Discovery A; Hologic).^19^ Physical function was assessed by the 400-m walk (aerobic capacity and walking endurance) and repeated chair rise (lower body muscle function),^20^ and upper and lower body muscle strength was assessed using 1-repetition maximum assessment for the chest press and leg press, respectively.^20^ Self-reported physical activity was assessed by the leisure score index from the Godin Leisure-Time Exercise Questionnaire.^21^ In addition, blood samples for prostate-specific antigen, testosterone, and C-reactive protein levels were collected and analyzed commercially by National Association of Testing Authorities–accredited laboratories in Australia.

### Statistical Analysis

Statistical analysis was performed from October 8 to December 23, 2024. The initial sample size calculation was based on detecting a moderate standardized effect (Cohen *d* = 0.5) in our primary as well as secondary outcomes of interest. To achieve 80% power at a 2-tailed α level of 0.05 and to account for an attrition rate of 20% or less, 80 patients per study arm were required for a total of 240 patients. However, due to slow recruitment and approval by the funding body to extend the research for an additional year, the trial management group closed recruitment at 112 patients December 20, 2018, before reaching target accrual. Analyses were conducted using SPSS Statistics, version 29 (IBM Corporation). Normality of distribution was assessed using the Kolmogorov-Smirnov test. Analysis of covariance (ANCOVA) adjusted for baseline values, age, current sexual activity, previous prostatectomy, previous radiotherapy, and previous or current ADT for primary and secondary outcomes. Data not normally distributed were log-transformed (ln) for analysis with ln(x +2) used for specific scales, as scores included zero. If exercise and exercise plus PESM were effective for improving sexual function in the specific domains assessed, then we tested for additional effects of exercise and PESM compared with exercise alone by using ANCOVA. Subgroup analyses were undertaken for patients treated with prostatectomy, previous or current radiotherapy, and previous or current ADT using ANCOVA adjusting for covariates used in the primary analyses. Trend analysis was performed using linear regression and entering tertiles of IIEF domains as an ordinal variable. Intention-to-treat analysis was used for maximum likelihood imputation of missing values (expectation maximization). Tests were 2 tailed with statistical significance set at *P* < .05.

## Results

Between July 24, 2014, and December 20, 2018, a total of 112 patients with prostate cancer (mean [SD] age, 66.3 [7.1] years) were randomized to exercise plus PESM (n = 36 [34.8%]), exercise only (n = 39 [32.1%]), or usual care (n = 37 [33.0%]) (Figure 1). Participant characteristics are presented in Table 1. Patients in the exercise plus PESM group attended 81% of scheduled exercise sessions and those in the exercise-only group attended 82% of the scheduled sessions. There were no major adverse events related to the exercise program, with only nonserious musculoskeletal-related adverse events reported (eTable 1 in Supplement 2).

**Table 1.** Patient Characteristics

Characteristics	Treatment group, No. (%) of patients		
	Exercise (n = 39)	Exercise plus PESM (n = 36)	Usual care (n = 37)
Demographic			
Age, mean (SD), y	66.5 (6.7)	64.6 (7.9)	67.8 (6.5)
BMI, mean (SD)	28.4 (3.9)	27.6 (3.7)	29.0 (5.0)
Married	30 (76.9)	26 (72.2)	28 (75.7)
Tertiary educational level	24 (61.5)	20 (55.6)	25 (67.6)
Currently employed	15 (38.5)	15 (41.7)	13 (35.1)
Godin LTEQ, median (IQR), score^a^^,^^b^	21.0 (9.0-30.0)	25.0 (16.0-35.0)	25.0 (12.0-36.8)
Clinical			
Time since diagnosis, median (IQR), mo^c^	10.0 (5.8-17.5)	9.0 (5.0-28.0)	8.0 (5.0-18.0)
No. of medications, median (IQR)	2.0 (1.0-3.0)	3.0 (1.0-4.0)	2.0 (1.0-5.0)
Other conditions			
Cardiovascular disease	5 (12.8)	5 (13.9)	5 (13.5)
Diabetes^d^	3 (7.9)	5 (13.9)	6 (16.2)
Dyslipidemia^d^	15 (38.5)	15 (41.7)	16 (44.4)
Hypertension^d^	15 (38.5)	21 (60.0)	18 (48.6)
Osteoporosis	2 (5.1)	2 (5.6)	0
PSA, median (IQR), ng/mL^d^	0.0 (0.0-0.3)	0.0 (0.0-1.1)	0.0 (0.0-0.3)
Testosterone, median (IQR), ng/dL^d^	221.9 (17.3-432.3)	89.3 (17.3-570.6)	155.6 (17.3-432.3)
Gleason score, median (IQR)^e^	7.0 (7.0-8.0)	7.0 (7.0-8.0)	8.0 (7.0-8.0)
Previous treatments			
Prostatectomy	22 (56.4)	20 (55.6)	21 (56.8)
Radiotherapy	12 (30.8)	12 (33.3)	12 (32.4)
Brachytherapy	4 (10.3)	3 (8.3)	1 (2.7)
ADT	16 (41.0)	18 (50.0)	19 (51.4)
Other treatments^c^	6 (16.2)	5 (14.3)	2 (5.6)
Current treatments			
Radiotherapy	10 (25.6)	3 (8.3)	8 (21.6)
Brachytherapy^d^	6 (15.4)	2 (5.7)	1 (2.7)
ADT	18 (46.2)	15 (41.7)	13 (35.1)
Other treatments^f^	5 (13.2)	3 (9.4)	2 (5.9)

Abbreviations: ADT, androgen deprivation therapy; BMI, body mass index (calculated as the weight in kilograms divided by the height in meters squared); LTEQ, Leisure-Time Exercise Questionnaire; PSA, prostate-specific antigen.

SI conversion factors: To convert PSA to μg/L, multiply by 1; testosterone to nmol/L, multiply by 0.0347.

^a^ Data were missing for 3 patients.

^b^ Moderate to strenuous LTEQ at least 24 was classed as active and 23 or less as insufficiently active.

^c^ Data were missing for 4 patients.

^d^ Data were missing for 1 patient.

^e^ Data were missing for 32 patients.

^f^ Data were missing for 8 patients.

### Sexual Function Outcomes

**Figure 2.**
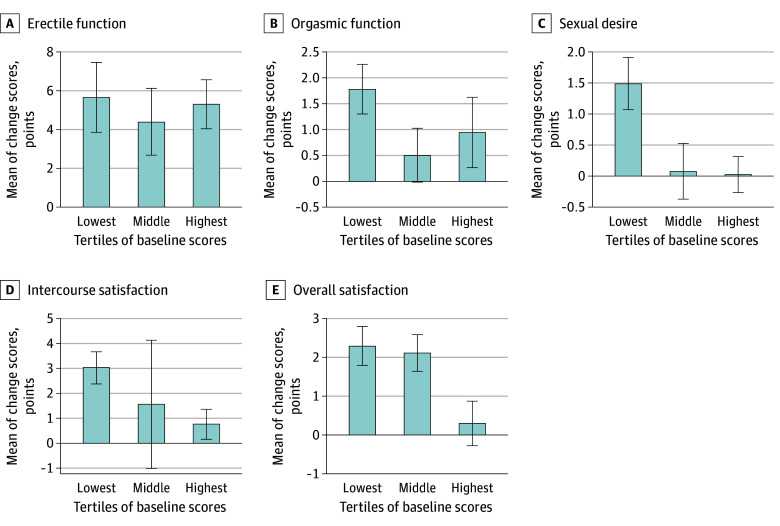
International Index of Erectile Function Domains Examined by Baseline Score Tertiles Patients with the lowest tertile scores prior to the initiation of exercise benefited the most following supervised exercise for sexual desire, intercourse satisfaction, and overall satisfaction.

Change in sexual function outcomes are provided in Table 2. The adjusted difference in IIEF erectile function scores at 6 months was in favor of exercise (5.1 points) compared with usual care (1.0 points; adjusted mean difference, 3.5; 95% CI, 0.3-6.6; *P* = .04). Change in intercourse satisfaction scores was not significant (adjusted mean difference, 1.7; 95% CI, 0.1-3.2; *P* = .05). When the intervention modalities were compared, PESM did not result in additional improvements in erectile function (adjusted mean difference, 1.1; 95% CI, −2.7 to 4.8; *P* = .89) or intercourse satisfaction (adjusted mean difference, −0.2; 95% CI, −2.1 to 1.6; *P* = .64). In subgroup analyses, the effects of exercise for erectile function were larger for the subgroups who received radiotherapy (adjusted mean difference, 4.2; 95% CI, 0.4-8.0; *P* = .11) and ADT (adjusted mean difference, 4.4; 95% CI, 0.2-8.7; *P* = .08) compared with the prostatectomy subgroup (adjusted mean difference, 1.6; 95% CI, −2.5 to 5.7; *P* = .36) (eTables 2-4 in Supplement 2). There was no statistically significant difference between exercise and usual care for sexual function assessed with the EPIC (adjusted mean difference, 7.9; 95% CI, 0.2-15.6; *P* = .09) or sexual activity assessed with the EORTC QLQ-PR25 (adjusted mean difference, 2.9; 95% CI, −4.1 to 9.9; *P* = .70) (Table 2), although based on the confidence intervals, some men would have experienced clinically relevant improvements. When the IIEF domains were examined by tertiles (Figure 2), those with the lowest tertile values prior to the initiation of exercise benefited the most following supervised exercise for sexual desire, intercourse satisfaction, and overall satisfaction.

**Table 2.** Sexual Health Outcomes

Outcome by treatment group	Mean (SD) score		Adjusted group difference, mean (95% CI)^a^	*P* value
	Baseline	Posttest		
IIEF erectile function^b^				
Exercise and exercise plus PESM	4.8 (4.8)	9.9 (9.1)	3.5 (0.3 to 6.6)	.04
Usual care	6.6 (7.0)	7.6 (9.1)		
IIEF orgasmic function^c^				
Exercise and exercise plus PESM	2.5 (2.8)	3.6 (3.4)	0.6 (−0.4 to 1.7)	.28
Usual care	2.4 (2.9)	2.9 (3.5)		
IIEF sexual desire^d^				
Exercise and exercise plus PESM	4.4 (2.1)	5.0 (2.3)	0.3 (−0.4 to 1.0)	.48
Usual care	4.3 (2.0)	4.6 (2.2)		
IIEF intercourse satisfaction^e^				
Exercise and exercise plus PESM	2.0 (3.1)	4.2 (4.6)	1.7 (0.1 to 3.2)	.05
Usual care	2.5 (3.7)	2.7 (4.0)		
IIEF overall satisfaction^d^				
Exercise and exercise plus PESM	3.6 (1.8)	5.1 (2.3)	0.4 (−0.5 to 1.3)	.30
Usual care	4.2 (2.0)	4.9 (2.5)		
EPIC sexual function^f^				
Exercise and exercise plus PESM	17.4 (16.4)	27.0 (22.9)	7.9 (0.2 to 15.6)	.09
Usual care	18.2 (17.1)	19.4 (21.7)		
EORTC QLQ-PR25 sexual activity^g^				
Exercise and exercise plus PESM	33.8 (23.1)	37.1 (22.8)	2.9 (−4.1 to 9.9)	.70
Usual care	31.1 (23.9)	32.4 (24.3)		

Abbreviations: EPIC, Expanded Prostate Cancer Index Composite; EORTC QLQ-PR25, prostate cancer module of the European Organisation for Research and Treatment of Cancer quality of life questionnaire; IIEF, International Index of Erectile Function; PESM, psychosexual education and self-management intervention.

^a^ Statistical analysis based on log(x) or log(x +2) transformed data.

^b^ Scores range from 1 to 30 with higher scores indicating better erectile function.

^c^ Scores range from 0 to 10, with higher scores indicating better orgasmic function.

^d^ Scores range from 2 to 10, with higher scores indicating greater sexual desire and greater overall satisfaction.

^e^ Scores range from 0 to 15, with higher scores indicating greater intercourse satisfaction.

^f^ Scores range from 0 to 100, with higher scores indicating better function.

^g^ Scores range from 0 to 100, with higher scores indicating higher levels of sexual activity.

### Body Composition, Physical Function and Strength, and Serum Markers

Change in body composition, physical function and strength, and blood markers are shown in Table 3. The adjusted mean difference for fat mass was −0.9 kg (95% CI, −1.8 to −0.1 kg; *P* = .02) at 6 months, favoring exercise compared with usual care, with no difference between groups for lean mass. Compared with usual care, exercise also significantly improved chair rise performance (adjusted mean difference, −1.8 seconds; 95% CI, −3.2 to −0.5 seconds; *P* = .002) and upper (adjusted mean difference, 9.4 kg; 95% CI 6.9-11.9 kg; *P* < .001) and lower (adjusted mean difference, 17.9 kg; 95% CI, 7.6-28.2 kg; *P* < .001) body muscle strength. There was no significant difference between groups for prostate-specific antigen, testosterone, or C-reactive protein levels.

**Table 3.** Body Composition, Physical Function and Strength, and Blood Markers

Outcome	Mean (SD) value		Adjusted group difference, mean (95% CI)^a^	*P* value
	Baseline	Posttest		
**Body composition**				
Fat mass, kg				
Exercise and exercise plus PESM	26.7 (8.2)	26.6 (8.3)	−0.9 (−1.8 to −0.1)	.02
Usual care	28.5 (9.7)	29.2 (9.7)		
Lean mass, kg				
Exercise and exercise plus PESM	56.1 (6.8)	56.7 (7.1)	0.1 (−0.6 to 0.7)	.81
Usual care	58.5 (8.0)	59.1 (8.1)		
**Physical function and strength**				
400-m walk, s				
Exercise and exercise plus PESM	232.9 (33.0)	226.7 (46.1)	−20.0 (−35.6 to −4.3)	.13
Usual care	240.1 (34.5)	252.9 (45.5)		
Chair rise, s				
Exercise and exercise plus PESM	10.7 (2.5)	10.1 (2.1)	−1.8 (−3.2 to −0.5)	.002
Usual care	11.9 (3.4)	12.6 (6.2)		
Chest press, 1-repetition maximum, kg				
Exercise and exercise plus PESM	46.7 (10.8)	56.4 (12.3)	9.4 (6.9 to 11.9)	<.001
Usual care	45.2 (9.7)	45.4 (9.9)		
Leg press, 1-repetition maximum, kg				
Exercise and exercise plus PESM	115.5 (42.2)	145.2 (50.6)	17.9 (7.6 to 28.2)	<.001
Usual care	114.0 (45.5)	124.9 (49.8)		
**Blood markers**				
PSA, ng/mL				
Exercise and exercise plus PESM	0.6 (1.4)	0.3 (0.8)	−0.4 (−1.1 to 0.4)	.30
Usual care	1.2 (3.7)	0.8 (3.3)		
Testosterone, ng/dL				
Exercise and exercise plus PESM	245.0 (256.5)	299.7 (247.8)	−5.8 (−74.9 to 60.5)	.82
Usual care	259.4 (282.4)	317.0 (265.1)		
CRP, mg/dL				
Exercise and exercise plus PESM	0.3 (0.4)	0.3 (0.3)	0 (−0.1 to 0.1)	.33
Usual care	0.4 (0.7)	0.2 (0.3)		

Abbreviations: CRP, C-reactive protein; PSA, prostate-specific antigen; PESM, psychosexual education and self-management intervention.

SI conversion factors: To convert CRP to mg/L, multiply by 10; PSA to μg/L, multiply by 1; testosterone to nmol/L, multiply by 0.0347.

^a^ Statistical analysis based on log(x) or log(x +2) transformed data.

## Discussion

This randomized clinical trial is, to our knowledge, the first exercise intervention study including brief psychosexual education with self-management for men with prostate cancer to examine sexual function as the primary outcome. As hypothesized, the supervised exercise program improved erectile function compared with usual care; however, there was no additional benefit of PESM. Moreover, when the IIEF domains were examined by tertiles, those with the lowest values prior to the initiation of exercise benefited the most following supervised exercise for sexual desire, intercourse satisfaction, and overall satisfaction. Exercise also had a significant effect on preventing gains in fat mass and resulted in significant improvements in physical function as well as upper and lower body muscle strength. These observations support the use of exercise as an effective intervention in the management of sexual dysfunction for men with prostate cancer.

Sexual dysfunction is a critical adverse effect of prostate cancer treatment and a major survivorship issue for patients and their partners. Exercise has been shown to improve patient-reported outcomes and reduce treatment toxicities, and is recommended in national and international cancer survivorship guidelines.^22,23^ However the evidence is less clear for sexual function in prostate cancer. In an unplanned post hoc analysis of a study examining short-term aerobic and resistance exercise on lean mass changes in patients undergoing ADT,^6^ patients in the exercise group preserved sexual activity, whereas the usual care group decreased it.^24^ This finding is supported by observational data showing that higher physical activity levels are associated with better sexual function in men with prostate cancer prior to radical prostatectomy and after external beam radiation therapy.^25,26^ Moreover, men with prostate cancer in the Health Professionals Follow-up Study who reported walking at a brisk pace (compared with an easy pace) had better sexual function, independent of walking duration.^27^ In the present study, exercise significantly improved erectile function (mean, 5.1 points) indicating a potentially clinically relevant improvement (minimal clinically important difference, 4.0 points)^28^ and resulted in higher intercourse satisfaction compared with usual care. A similarly positive effect on erectile function was observed in a study comparing yoga with usual care in men with prostate cancer during radiotherapy, where yoga prevented a decline in erectile function at 4 (but not 8) weeks of treatment.^29^ Further, in middle-aged and older men, aerobic exercise has been shown to improve erectile function with a mean change in the IIEF domain of 2.8 points.^30^ In contrast, equivalent benefits of exercise on erectile function were not observed in a study of aerobic training after radical prostatectomy^31^ or in men with advanced prostate cancer.^32^ It may be that timing of exercise implementation, exercise mode, or stage of disease and accumulated effects of treatments account for these differences. However, an important observation from our trial was that patients with the lowest values in sexual desire, intercourse satisfaction, and overall satisfaction prior to the initiation of exercise benefited the most following supervised exercise. Regaining sexual function is not a rehabilitation goal for all men with prostate cancer and varies according to relationship status, comorbid health conditions for both the man and their partner, and personal priorities and values. Screening patients for sexual dysfunction and rehabilitation goals following treatment could assist directing patients to exercise as a countermeasure, forming part of an accessible evidence-based survivorship intervention.^33^

Our low-intensity psychoeducation intervention had no additional effect on sexual function outcomes in the present study. We hypothesized that a brief PESM intervention would further enhance improvements in sexual function by increasing the participants’ ability to better self-manage their well-being and sexual function through, for example, increased uptake of pharmacological management for erectile dysfunction. Chambers et al^11^ previously reported that multimodal psychosocial and/or psychosexual interventions are shown to improve mental health and quality of life, as well as increase sexual satisfaction and decrease sexual bother in men with prostate cancer. However, a recent online psychosexual support intervention for couples after prostate cancer treatment did not improve global sexual satisfaction, although couples who received the intervention did engage in more sexual activity.^34^ Our psychosexual support delivered by the exercise physiologist as part of a PESM adjunctive component may not have been powerful enough to improve outcomes above the exercise intervention effect. Given the impact treatments have on erectile function, a more intense intervention that targets adherence to medical management of erectile dysfunction^35^ as well as the couple relationship^36^ might be indicated.

As expected, the exercise intervention resulted in significant improvements in physical function and muscle strength and prevented an increase in fat mass, which occurred in the usual care group. Exercise for men with prostate cancer is an established intervention to address treatment-related deterioration in these outcomes,^6,37^ which can negatively impact sexual function and quality of life.^8^ Meeting physical activity recommendations for aerobic exercise has been associated with significantly better masculine self-esteem in men with prostate cancer, which was strongly correlated with perception of body image in this group of men,^38^ both important factors contributing to sexual function. Exercise, specifically resistance training, can therefore play an active part in sexual function by counteracting treatment-related changes in body composition, physical function, and muscle strength.^9^ Further, despite well-established reductions in treatment adverse effects and improvements in quality of life^6,39,40^ and an association of exercise and prostate cancer survival,^41^ many men with prostate cancer remain insufficiently active.^42^ Our present study potentially provides an additional rationale for taking up exercise for men who are concerned about their sexual function.

### Strengths and Limitations

Our study has several features that are worthy of comment. First, we investigated a highly significant outcome of sexual function in men with prostate cancer who were concerned about their sexual function and were within 12 months of treatment. Second, we compared usual care and exercise with and without PESM support. Furthermore, participants had high adherence to the exercise intervention, reflecting the program’s overall feasibility and effectiveness to produce favorable outcomes in body composition, physical function, and muscle strength.

Nevertheless, this study also has some limitations. The trial was originally designed as a multisite study (in 3 states in Australia); however, due to logistical issues it was modified to be a single-center study (in Western Australia). In addition, recruitment difficulties resulted in the study management group closing recruitment at 112 patients before reaching target accrual and, as such, the study was likely underpowered. Our patients were well-functioning individuals who were motivated to undertake the intervention program and the supervised exercise sessions and may not be representative of all men with prostate cancer.

## Conclusions

In this randomized clinical trial, we found that supervised resistance and aerobic exercise improved erectile function and intercourse satisfaction in men with prostate cancer previously or currently undergoing treatment, although the addition of psychosexual education resulted in no additional improvements. Based on the findings of this study, exercise should be considered as an integral part of treatment to improve sexual function in men with prostate cancer.

## Notes

Supplement 1.
Trial Protocol

Supplement 2. **eTable 1.** Summary of Adverse Events (Baseline to 6 Months) **eTable 2.** Subanalyses: Prostatectomy Cohort **eTable 3.** Subanalyses: Radiotherapy Cohort **eTable 4.** Subanalyses: ADT Cohort

Supplement 3.
Data Sharing Statement
