# Supplementary material for: Exercise and Psychosexual Education to Improve Sexual Function in Men With Prostate Cancer: A Randomized Clinical Trial
Source: JAMA Netw Open. 2025 Mar 12;8(3):e250413. doi: 10.1001/jamanetworkopen.2025.0413 (PMC11904736; doi:10.1001/jamanetworkopen.2025.0413)
Supplement: Supplement 1. — Trial Protocol [file jamanetwopen-e250413-s001.pdf]

Sexual Health Clinical Study Protocol

IMPROVING SEXUAL HEALTH IN MEN WITH PROSTATE CANCER: RANDOMISED CONTROLLED TRIAL OF EXERCISE AND PSYCHOSEXUAL THERAPIES.

Protocol Number:	1
Investigational Product:	Aerobic and resistance training; psychosexual education
Funder:	National Health and Medical Research Council

Status: Final Protocol
Date: January 27, 2014
Prepared by: Cailyn Walker
Document No.: v1.0

GCP Compliance: This study will be conducted in compliance with Good Clinical Practice, and applicable regulatory requirements.

The confidential information in the following document is provided to you as an investigator, potential investigator, or consultant, for review by you, your staff, and appropriate ethical review committee. By accepting this document, you agree that the information contained herein will not be disclosed to others without written authority from Edith Cowan University, except to the extent necessary to obtain approval of this protocol by an ethical review committee.

PROTOCOL AGREEMENT

I confirm that I have read this protocol. I will comply with the protocol and the principles of Good Clinical Practice (GCP) and applicable regulatory requirements.

Protocol: **v1.0**

Protocol Title: **Improving sexual health in men with prostate cancer: randomised controlled trial of exercise and psychosexual therapies.**

____ ____________________

Investigator Signature *Date*

____

Print Name and Title

Site # ____________

Site Name ____

PROTOCOL SYNOPSIS

**Title**	**Improving sexual health in men with prostate cancer: randomised controlled trial of exercise and psychosexual therapies.**
**Funder**	National Health and Medical Research Council
**Study Design**	Randomised Controlled Trial
**Primary Objective**	To examine the efficacy of exercise as a therapy to aid in the management of sexual dysfunction in men with prostate cancer.
**Secondary Objectives**	▪ To determine if combining exercise and psychosexual intervention results in more pronounced improvements in the sexual health of men with prostate cancer.<br>▪ To assess if any benefit of exercise and psychosexual intervention on sexual dysfunction in men with prostate cancer is sustained long term.
**Number of Patients**	240
**Inclusion Criteria**	▪ Concerned about their sexual health as assessed by International Index of Erectile Functioning (IIEF) overall satisfaction score <8 (i.e. moderately-very dissatisfied) and/or Expanded Prostate Cancer Index Composite (EPIC) sexual bother score >8 (i.e. small-big problem).<br>▪ Prior-current treatment for prostate cancer including prostatectomy, radiotherapy or AST.<br>▪ Medical clearance to participate in the project.
**Exclusion Criteria**	▪ Non-nerve sparing prostatectomy.<br>▪ >6 months since prostatectomy or completion of radiotherapy or AST.<br>▪ Incontinence defined as requiring the use of >1 pad in a 24-hour period.<br>▪ Acute illness or any musculoskeletal, cardiovascular or neurological disorder that could inhibit exercise performance or put participants at risk from exercising.<br>▪ Men who are already performing regular exercise, defined as undertaking structured resistance and aerobic training two or more times per week within the past 3 months, are not eligible.<br>▪ Unable to read and speak English.
**Safety Assessments**	Blood pressure and heart rate recorded at every assessment (blood pressure should be monitored at every training session if high during assessments (>160/95mmHg). Adverse events collected continuously from baseline until end of 2 year follow up assessment.

**Primary Endpoint**	The IIEF will be utilized to assess sexual health across a variety of domains including erectile function, orgasmic function, sexual desire, intercourse satisfaction and overall satisfaction. The Sexual Function scale of the EPIC is a prostate cancer specific tool which will be utilized to assess sexual function and satisfaction. Sexual activity level will be assessed using the sexual domain of the European Organisation for Research and Treatment of Cancer (EORTC) prostate cancer specific questionnaire (QLQ-PR25).
**Secondary Endpoints**	Secondary outcomes include key factors associated with sexual health in men with prostate cancer.<br>▪ Sexual Self-Confidence. The Short Form Psychological and Interpersonal Relationship Scale will be used to assess sexual self-confidence.<br>▪ Masculine Self-Esteem. The Masculine Self-Esteem Scale is designed specifically for use in men with prostate cancer and will be utilised to assess participant’s appraisal of their masculinity.<br>▪ Utilisation of Sexual Aids. A previously developed scale will assess whether participants have sought medical assistance for sexual dysfunction at any point before, during or after prostate cancer treatment. All treatments will be documented and the impact of these treatments rated.<br>▪ Relationship Satisfaction. The Dyadic Adjustment Scale will assess relationship satisfaction between participants and their partners.<br>▪ Sexual Supportive Care Needs. The level of need for help with sexual dysfunction will be assessed by the sexuality domain of the Supportive Care Needs Survey.<br>▪ Quality of Life. Health-related quality of life will be assessed using the Medical Outcomes Short Form 36 (SF-36). Quality of life will also be assessed using a prostate cancer specific tool, the EORTC QLQ-C30 and QLQ-PR25 questionnaires.<br>▪ Urinary, Bowel & Hormonal Issues. Adverse side effects specific to prostate cancer treatments will be assessed using the urinary function, bowel habits and hormonal function scales of the EPIC.<br>▪ Psychological Distress. The Brief Symptom Inventory-18 will be utilised to assess psychological distress across the following domains: anxiety, depression, somatisation and global distress severity. Antidepressant use will also be recorded.<br>▪ Fatigue. Cancer related fatigue will be assessed using the Functional Assessment of Chronic Illness Therapy-Fatigue questionnaire.<br>▪ Body Composition. Regional and whole body lean mass and fat mass will be derived from whole body dual-energy X-ray absorptiometry scans. Trunk adiposity, visceral fat and adipose indices will be assessed using standard procedures.

	- Body Image. The Body Image Scale is a cancer-specific scale that will be used to assess participants perceptions of their appearance.<br>- Physical Function & Physical Activity Levels. A series of standard tests will be used to assess physical function:<br>o 400-m walk (aerobic capacity).<br>o one repetition maximum in the leg press and chest press (muscular strength).<br>o repeated chair rise (muscular power).<br>o usual and fast pace 6-m walk (ambulation).<br>o backwards tandem 6-m walk (balance).<br>o Physical activity levels will be assessed objectively over a 7-day period using a validated, reliable tri-axial accelerometer activity monitor (ActiGraph GT3X+).<br>o Self-reported physical activity will also be assessed by the leisure score index from the Godin Leisure-Time Exercise Questionnaire.<br>Blood Biomarkers. Blood samples will be collected and analysed commercially by accredited Australian National Association of Testing Authorities laboratories for testosterone, high sensitivity C-reactive protein (CVD risk; also related to severity of penile vascular disease) and PSA.
**Rationale for Number of Patients**	Data from our 3-month study in prostate cancer patients indicates that the standard deviation for change in our primary outcome of sexual health equates to ~22 points. We observed the mean change after the intervention to differ between exercise and usual care groups by ~11 points[1]. There is no available evidence comparing changes in sexual health between exercise and psychosexual interventions. However, data from previous psychosexual interventions in prostate cancer patients indicates changes of a moderate standardised effect (d = 0.5) in sexual health following the psycho-educational interventions[2,3]. We have based sample size calculations on the assumption that there will be an additive effect of exercise and psychosexual therapies. A priori, 64 participants per group will be required to achieve 80% power at an alpha level of 0.05 (two tailed), and to demonstrate a difference between the three groups in sexual health at the end of the intervention. Previous experience in exercise and psychosexual therapy trials indicates an attrition rate of up to 20% over the intervention period. Therefore, to adequately ensure that we have sufficient participant numbers at the end of the intervention, 240 participants will be randomised to the study arms. A sample size of 240 will also provide us with sufficient power to detect a moderate standardised effect (d = 0.5) in our secondary outcomes. Based on previous experience we anticipate a further attrition rate of up to 20% over the follow-up period. Therefore, the sample size will provide 87% statistical power to detect differences between the exercise group and the exercise + psychosexual group at follow-up<br>(d = 0.5; n = 77 per group as the usual care group will be split between the two interventions for the prospective cohort study component of this project).

**Statistical Analysis Plan**	Analyses will be conducted using IBM SPSS Statistics (latest version) (IBM corp., Armonk, NY) with an intention-to-treat approach using maximum likelihood imputation of missing values (expectation maximization). Normality of the distribution will be assessed using the Kolmogorov-Smirnov test. Analyses will include standard descriptive statistics, chi-square, correlation and regression, and analysis of variance (ANOVA) or covariance (ANCOVA). Differences at baseline will be assessed using one-way analysis of variance (ANOVA) or the Kruskal-Wallis test, as appropriate, for continuous data and Chi-square for categorical data. For the primary and secondary outcomes, a two-way (group x time) repeated-measures ANOVA or ANCOVA, as appropriate, will be undertaken. Follow-up tests will be performed if the interaction or the main effect of time is significant. Data not normally distributed will be log transformed (ln) for analysis. Initially the exercise and exercise plus self-managed psychosexual therapy will be combined and compared to usual care. If exercise and exercise plus self-managed psychosexual therapy are effective for improving sexual health, then the additional effects of exercise and self-managed psychosexual therapy will be compared to exercise alone by using a two-way (group x time) repeated-measures ANOVA (or ANCOVA as appropriate). To examine the effect of the intervention based on baseline sexual function, trend analysis will be performed using linear regression and entering tertiles of the sexual health domains as an ordinal variable. All tests will be two-tailed with an alpha level of 0.05 for statistical significance.
**Duration of Patient Participation and Duration of Study**	Patient participation: 18 months<br>Duration of Study: 48 months

LIST OF ABBREVIATIONS

**AE**	adverse event
**AEP**	accredited exercise physiologist
**ANOVA**	analysis of variance
**ANCOVA**	analysis of covariance
**AST**	androgen suppression therapy
**CRP**	C-reactive protein
**CVD**	Cardiovascular disease
**DXA**	Dual-energy X-ray Absorptiometry
**EORTC**	European Organisation for Research and Treatment of Cancer
**EPIC**	Expanded Prostate Cancer Index Composite
**GCP**	good clinical practice
**HREC**	Human Research Ethics Committee
**IIEF**	International Index of Erectile Functioning
**NIH**	National Institute of Health
**NHMRC**	National Health and Medical Research Council
**NSW**	New South Wales
**PCa**	prostate cancer
**PDE-5**	Phosphodiesterase-5
**PSA**	prostate-specific antigen
**QLD**	Queensland
**QLQ-PR25**	prostate cancer specific questionnaire
**QLQ-PR30**	cancer specific questionnaire
**QOL**	quality of life
**RCT**	randomized controlled trial
**RM**	repetition maximum
**1RM**	one repetition maximum
**SAE**	serious adverse event
**SF-36**	Medical Outcomes Short Form 36
**WA**	Western Australia

TABLE OF CONTENTS

1.0 INTRODUCTION ______ *9*

2.0 RESEARCH PLAN ______ *12*

3.0 INCLUSION CRITERIA ______ *13*

4.0 EXCLUSION CRITERIA ______ *13*

5.0 SCREENING, RANDOMIZATION and STRATIFICATION ______ *13*

6.0 CLINICAL PROCEDURES ______ *14*

7.0 EXERCISE INTERVENTION ______ *18*

8.0 EXERCISE AND PSYCHOSEXUAL INTERVENTION ______ *19*

9.0 USUAL CARE ______ *19*

10.0 STATISTICAL CONSIDERATIONS ______ *19*

11.0 ADVERSE EVENTS AND REPORTING REQUIREMENTS ______ *20*

12.0 WITHDRAWAL OF PARTICIPANTS ______ *22*

13.0 DATA MANAGEMENT AND MONITORING ______ *22*

14.0 AMENDMENTS ______ *22*

15.0 REFERENCES ______ *22*

16.0 APPENDICES ______ *27*

1.0 INTRODUCTION

1.1 Background

Sexual dysfunction secondary to cancer treatment is a primary concern of men with prostate cancer and the most frequently identified issue of importance among this patient population[4,5]. Men who receive treatment for prostate cancer through either radical prostatectomy, radiotherapy (including external beam and brachytherapy) or androgen suppression therapy (AST) suffer significant sexual dysfunction[6-14]. Compared to their age-matched counterparts, men with prostate cancer have a 10-15 fold increased prevalence of erectile dysfunction[15]. The incidence of erectile dysfunction amongst men with prostate cancer is reported to range from ~14 to ~90% (the variability is likely due to differences in assessment tools, types of treatment and timing of assessment)[7,8]. A large percentage of men with prostate cancer also experience loss of libido (~60-90%), penile shortening (~68%) and altered orgasm experience (~64-87%)[7,8,14]. These issues culminate in a high incidence of overall sexual dissatisfaction in men with prostate cancer (~61-91%)[7,8,14]. Not only is sexual dysfunction highly prevalent among prostate cancer survivors but the degree of impairment is significant, with ratings of sexual health decreasing to ~17-55% of pre-treatment levels two years after treatment[6,9,11]. Furthermore, compared to otherwise healthy men with erectile dysfunction, prostate cancer survivors with erectile dysfunction have significantly lower levels of erectile function, intercourse satisfaction, orgasmic function and sexual self-efficacy[16]. While it is unclear what percentage of men undergoing radiotherapy or AST return to pre-treatment levels of sexual health, only ~31% of men return to pre-treatment levels following prostatectomy and this restoration does take a considerable amount of time (2-3 years)[13]. It is clear that sexual dysfunction is a highly prevalent and enduring adverse effect of prostate cancer treatment[7,8,14]. Given that sexuality is a fundamental component of being a human being, it is not surprising that sexual dysfunction has a significant impact on quality of life in prostate cancer survivors and their partners[4,6-8,14,16,17]. These data highlight that sexual dysfunction is a critical survivorship care issue.

1.2 Aetiology of Sexual Dysfunction Following Prostate Cancer Treatment

Sexual dysfunction is a complex, multidimensional health issue that incorporates physiological, psychological and interpersonal elements. Hence, the causes of sexual dysfunction are multifaceted. In otherwise healthy men, the primary factors contributing to sexual dysfunction are most commonly age-related declines in androgen levels and changes in erectile function[18] as well as chronic diseases (e.g. cardiovascular disease [CVD], diabetes, obesity, depression)[18-20]. The treatment of prostate cancer compounds these factors through a number of avenues. Prostatectomy and radiotherapy can result in structural damage to erectile nerves and/or blood vessels; subsequent denervation and/or ischemic processes can lead to fibrosis of the corpus cavernosa and cause erectile dysfunction[7,8]. These treatments can also result in penile shortening, reduced/absent ejaculation, altered orgasm and reduced libido[7,8]. Testosterone ablation through AST leads to erectile dysfunction, loss/elimination of libido, penile shortening, testicular atrophy, reduced/absent ejaculation, altered or painful orgasm, body feminisation (loss of muscle mass, weight gain, loss of body hair, gynecomastia), body image dissatisfaction, fatigue, reduced physical function and emotional lability[7,8,14,21]. AST is also associated with an increased risk of CVD, diabetes and obesity, all of which are associated with compromised sexual health[22-24]. Additionally, depression, anxiety and reduced quality of life are common across treatment modalities and these factors further contribute to sexual dysfunction[7,8,14]. Collectively, these issues are highly emasculating because they compromise core components of male identity such that men with prostate cancer no longer feel or function fully as men[7,17,25,26]. This loss of masculinity can subsequently contribute to further sexual dysfunction in prostate cancer survivors as reflected in the observation of masculine self-esteem as an independent predictor of sexual bother[25,27]. Furthermore, the impact of prostate cancer on intimate relationships (e.g. inability to maintain usual sexual practices) can also contribute to sexual dysfunction[7,14,17]. The multifaceted aetiology of sexual dysfunction following prostate cancer treatment dictates that a comprehensive approach must be employed to effectively manage this condition.

1.3 Management of Sexual Dysfunction Following Prostate Cancer Treatment

Despite the clear need for effective management of sexual dysfunction and calls for incorporation into standard survivorship care, there is a scarcity of knowledge regarding evidence-based intervention[7,28,29]. The majority of research has focused on early penile rehabilitation after prostatectomy through the use of phosphodiesterase-5 (PDE-5) inhibitor drugs, which is considered first-line therapy for treatment of erectile dysfunction[7,8,28,29,30].

PDE-5 inhibitors have established efficacy for improving erectile function following prostatectomy and/or radiotherapy[7,8,28,29,30]. Second-line therapy commonly involves the use of vacuum constriction devices and penile injections with intracavernosal vasodialators, followed by penile implant surgery as a third-line therapy option[7,29,30]. While there is research indicating benefit in men without cancer[31,32], there is insufficient evidence to determine the efficacy of these secondary interventions in prostate cancer survivors[28]. Furthermore, the uptake, compliance and satisfaction with all of these treatment options are relatively low[33-35]. For example, only 33-44% of prostate cancer survivors experiencing sexual dysfunction who tried these treatments found them to be at least somewhat helpful[33]. These treatment options are also provided following AST but fail to address many of the issues specific to AST such as loss/elimination of libido and body feminisation[7]. Herein lies a fundamental flaw in the current clinical management of sexual dysfunction in prostate cancer survivors; pharmacological intervention only focuses on counteracting declines in erectile function and no strategies exist to address the multifaceted aetiology of sexual dysfunction. Significant sexual dysfunction is apparent 1-2 years after prostatectomy in men who regain erectile function[36], highlighting the need for more comprehensive management approaches. Incorporation of psychosexual interventions as a first-line therapy have been suggested to address psychological and interpersonal elements involved with compromised sexual health[7,28,30,37]. Current evidence indicates that theoretically based psychosexual interventions involving patient, couple or group education and counselling targeting psychosocial and sexual health are moderately effective in counteracting sexual dysfunction secondary to cancer[37,38]. In direct contrast to the scale of the problem, relatively little research has focused on men with prostate cancer therefore insufficient evidence is available to guide optimal psychosexual therapy specific to this patient population[28]. Importantly, psycho-oncologic interventions have established efficacy for improving psychological distress and quality of life in cancer patients[39], factors which contribute to sexual dysfunction following cancer treatment[7,8,17,40]. While pharmacological and psychosexual interventions aid in the management of sexual dysfunction, there are numerous treatment sequelae which compromise sexual health that simply aren't addressed with these therapies (e.g. loss of libido, body feminisation, fatigue, co-morbid conditions).

1.4 Exercise Therapy: An Innovative Approach to the Management of Sexual Dysfunction

Considerable research has established the efficacy of exercise in mitigating many adverse treatment related side effects of prostate cancer including factors central to the aetiology of sexual dysfunction[22,41-44]. Specifically, prostate cancer survivors involved in exercise therapy have significant improvements in body composition, fatigue, physical function, depression, anxiety and quality of life as well as a reduction in the risk of co-morbid conditions (i.e. reduced risk factors for CVD, diabetes, obesity and osteoporosis)[41-44]. While the direct effect of exercise on sexual dysfunction in prostate cancer survivors has yet to be examined, physical activity level is independently associated with sexual dysfunction in men without prostate cancer[15,18,19,45-48]. Specifically, exercise has a protective effect against erectile dysfunction in that less active men have a ~40-60% greater risk of erectile dysfunction compared to more active men (after multivariate adjustments for confounders)[15,18,19,45-48]. Similar results have been observed in prostate cancer survivors following radiotherapy, with greater levels of physical activity significantly associated with superior sexual health (after controlling for age, medical co-morbidity, fatigue and urinary and bowel function)[40]. We know of no research investigating the impact of supervised exercise therapy on sexual health, however, interventions involving advice about positive exercise behaviour as part of a lifestyle modification program have reported improved sexual health in obese men[49] as well as men with, or at risk of erectile dysfunction[50]. Importantly, changes in sexual health were independently associated with changes in physical activity levels[49]. Men who suffer from erectile dysfunction are therefore advised to increase their physical activity level[31,32] but this evidence hasn't translated into similar recommendations for prostate cancer survivors experiencing sexual dysfunction. Despite this, sound theoretical rationale suggests that exercise may represent an effective therapy to enhance sexual health following prostate cancer treatments.

1.5 Preliminary Results

Our team has identified the adverse effects of prostate cancer treatment on sexual health as well as factors associated with sexual dysfunction including musculoskeletal toxicities, physical function decline, psychological distress, fatigue, pain, increased risk of co-morbid conditions and reduced quality of life[11,51-57]. As a countermeasure strategy we have reported the considerable beneficial effects of exercise in attenuating these adverse side effects (including national and international exercise guidelines for cancer patients)[1,3,42-44,58-67,], an

area of ongoing study for our team[57,62,68,69]. Furthermore, our team has identified unmet psychosocial supportive care needs of prostate cancer patients[70-76]and developed psychosexual interventions to counteract distress in survivors[77-80] another area of ongoing research for our team[81-84]. This work has led us to perform a series of initial investigations to explore the role of exercise and psychosexual therapies in managing sexual dysfunction.

In a secondary analysis of data from a RCT published in the Journal of Clinical Oncology we evaluated the impact of exercise on sexual health (n = 57; treatment: 100% current AST, 63% previous/current radiotherapy, 40% previous prostatectomy)[1]. Sexual activity (representing libido and the level of sexual activity with or without intercourse) did not differ between exercise and usual care groups at baseline but there was a significant group difference following the 3-month intervention (exercise > usual care; group difference = 11.7 points on the QLQ-PR25, p = 0.045). This difference was primarily driven by changes in libido. While the percentage of participants with high libido was similar between groups at baseline (exercise = 20.6%; usual care = 22.2%), the percentage of participants with high libido was significantly greater in the exercise group following the intervention (exercise = 17.2%; control = 0%; p = 0.024). The protective effect of exercise on sexual activity was theorised to be mediated by improvements in quality of life as significant relationships were observed between the change in sexual activity and the change in quality of life domains general health (rho = 0.335, p = 0.011) and role-emotional (rho = 0.295, p = 0.030). Furthermore, the exercise intervention also resulted in significant improvements in factors associated with sexual dysfunction, namely body composition, muscle strength, aerobic capacity, physical function and vitality as well as a significant reduction in fatigue[43]. These improvements may have contributed to the superior sexual activity scores observed in the exercise group. This theory is supported by our subsequent investigation involving 163 prostate cancer patients (treatment: 100% current AST, 36% previous radiotherapy, 21% previous prostatectomy) in our recently completed NHMRC funded RCT (ID#534409)[57]. Although not the primary purpose of the trial, we observed sexual activity to improve by 8.9% following 6 months of exercise therapy compared to a 14.8% decrease following usual care (group difference = 4.0 points on the QLQ-PR25, p = 0.150). The relationship between the change in sexual activity during the intervention was significantly related to the change in factors commonly associated with sexual dysfunction including quality of life (general health, physical function, vitality, role-emotional, social function and body pain domains), body composition, lower body muscular strength and psychological distress (somatisation). Although these trials were not designed to address sexual dysfunction directly, the findings indicate that exercise is a promising therapy for the management of sexual dysfunction following prostate cancer.

We theorise that a key mechanism contributing to the exercise induced maintenance/ improvement in sexual health may be the ability of exercise to foster improved feelings of masculinity in men with prostate cancer. To explore this theory, we performed a qualitative study addressing issues surrounding sexual health concerns during prostate cancer treatment and the potential role of exercise (n = 10; treatment: 100% current AST, 30% previous radiotherapy, 20% previous prostatectomy). Concerns about masculinity and body image emerged as themes contributing to distress about their sexual health (e.g. “I felt de-masculinised in a sense and well I was fairly impotent at the time”; “I felt that I was going to turn into a little ball of fat”). The role of exercise in improving physical and mental wellbeing as well as the peer support provided by the group exercise sessions were themes which emerged as contributing factors in coming to terms with sexual dysfunction. However, reinforcement of masculinity through exercise was the strongest theme to emerge from the analyses. Participants felt that “the exercise itself has actually made me feel more masculine” through improving perceptions of their body image (e.g. “while exercising I was really noticing that I was keeping the weight off and getting my muscle tone back”) as well as engagement in a masculine activity shared with other men (e.g. “I was doing manly things, sweating it out with a whole bunch of other guys”). Masculinity has also been observed to be a critical factor in the level of psychological distressed experienced by men with prostate cancer. We performed analyses on the baseline data from our recent NHMRC funded (ID#496001) psychosexual therapy RCT which involves 189 men (treatment: 100% prostatectomy) and their partners[80]. Masculine self-esteem was significantly related to psychological distress in prostate cancer patients and was the strongest predictor of depression (19% of the variance), anxiety (13% of the variance) and mental quality of life (14% of the variance). Furthermore, the patient’s psychological distress and his sexual bother were significantly related to his partner’s mental health status, with male psychological distress the strongest predictor of partner depression (6% of the variance). There were no interactions between psychological distress and intimacy for either the patients or their partners, suggesting that drivers of distress in men with

prostate cancer are internal rather than relationship focussed. This observation is supported by a previous report that involvement of the partner in a psychosexual intervention does not impact the ability of the intervention to improve sexual health in men with prostate cancer[2]. Thus, these data indicate that masculine self-esteem is central to the distress associated with prostate cancer and suggests that interventions should target re-building feelings of masculinity. It also appears that masculinity is a construct that is amenable to change through exercise.

Despite being a critical survivorship care issue, there is a clear gap in current knowledge of the optimal treatment of sexual dysfunction in men with prostate cancer. The proposed study will generate information to address this gap. There is a strong theoretical rationale and emerging evidence that exercise is an innovative therapy to counteract sexual dysfunction in cancer survivors. However, no previous research has investigated the efficacy of exercise on sexual health following cancer treatment. Furthermore, despite the multidimensional aetiology of sexual dysfunction, there is a paucity of research investigating the efficacy of integrated treatment models. The proposed study will address these limitations. Findings from this study will expand current clinical guidelines for the management of sexual dysfunction in men with prostate cancer and importantly facilitate the development of targeted supportive care services for survivors concerned by their sexual health. Evidence gained may lead to a paradigm shift in the management of sexual dysfunction in prostate cancer survivors.

2.0 RESEARCH PLAN

2.1 Aims

To characterise and quantify the benefits of exercise and psychosocial therapies on sexual health for men with prostate cancer, we propose to undertake a multi-centre randomised controlled trial (RCT) of 6 months supervised resistance and aerobic exercise ± psychosexual intervention versus usual care in 240 men with PCa, with subsequent follow-up over 1 year to examine the long-term impact of exercise and psychosexual therapies on sexual dysfunction in prostate cancer patients.

Aims:

1. Examine the efficacy of exercise as a therapy to aid in the management of sexual dysfunction in men with prostate cancer.
2. Determine if combining exercise and psychosexual therapies results in more pronounced improvements in the sexual health of men with prostate cancer.
3. Assess if any benefit of exercise and psychosexual therapies on sexual dysfunction in men with prostate cancer is sustained long term.

We will evaluate the following hypotheses:

1. Compared to usual medical care, exercise will improve sexual health in men with prostate cancer who are concerned by sexual dysfunction. We theorise that exercise will improve masculine self-esteem, quality of life, psychological distress, fatigue, body composition, body image and physical function, culminating in increased sexual health.
2. When exercise and psychosexual therapies are combined, improvements in sexual health will exceed those observed in usual medical care and exercise therapy. We theorise that psychosexual therapy will further enhance improvements in sexual health through additional improvements in masculine self-esteem, psychological distress and quality of life as well as increased utilisation of sexual aids (i.e. enhanced uptake of pharmacologic management).
3. Improvements in sexual health will be sustained 1 year after completion of the exercise and combined exercise and psychosexual interventions. We hypothesise that the theoretically based interventions will prompt behavioural change that leads to sustained improvements in sexual health.As a secondary hypothesis we propose that the effect of exercise on psychological outcomes and mental QOL will be mediated by masculine beliefs and self-efficacy.

2.2 Study Design

A single-blinded (investigators blinded to group allocation), three arm, multi-site RCT design will be used to

examine the efficacy of exercise and psycho-sexual therapies on sexual health in men with prostate cancer. An ‘Exercise’ group (1) will complete the exercise intervention, an ‘Exercise + Psychosexual’ group (2) will complete the same exercise intervention as well as a psychosexual intervention and a ‘Usual Care’ group (3) will maintain usual medical care for a period of 6 months. The Usual Care group will be offered participation in the interventions at the completion of the 6-month period (half to receive the Exercise intervention and half the Exercise + Psychosexual intervention). The RCT will be followed by a prospective cohort study examining the long-term impact of exercise and psychosexual therapies on sexual dysfunction in prostate cancer patients 1 year after the interventions (Table 1). The study will be guided by the CONSORT statement[85].

Table 1. Study Design (note: self-report measures also assessed at 6 months follow up).

Months	0	6	12	18	24
**(1) EXERCISE THERAPY**	Intervention	1 year Follow-up			
**(2) EXERCISE + PSYCHO-SEXUAL THERAPIES**	Intervention	1 year Follow-up			
**(3) USUAL CARE**	Usual care	Intervention	1 year Follow-up		

3.0 INCLUSION CRITERIA

3.1 Concerned about their sexual health as assessed by International Index of Erectile Functioning (IIEF) overall satisfaction score < 8 (i.e. moderately-very dissatisfied)[86] and/or Expanded Prostate Cancer Index Composite (EPIC) sexual bother score > 8 (i.e. small-big problem)[87];

3.2 Prior/current treatment for prostate cancer including prostatectomy, radiotherapy or AST; and

3.3 Medical clearance to participate in the project.

4.0 EXCLUSION CRITERIA

4.1 Non-nerve sparing prostatectomy;

4.2 >6 months since prostatectomy or completion of radiotherapy or AST;

4.3 Incontinence defined as requiring the use of >1 pad in a 24-hour period;

4.4 Acute illness or any musculoskeletal, cardiovascular or neurological disorder that could inhibit exercise performance or put participants at risk from exercising;

4.5 Men who are already performing regular exercise, defined as undertaking structured resistance and aerobic training two or more times per week within the past 3 months, are not eligible; or

4.6 Unable to read and speak English.

5.0 SCREENING, RANDOMIZATION AND STRATIFICATION

5.1 Screening

Patients that are potentially eligible for the project should be given the information leaflet from their treating physician and asked if they are interested in learning more about the study. Interested patients will then be referred to the research team. Patients who have been referred will be contacted by a research team member at the patients’ local sites to tell the patients more about the study, answer any questions, assess interest, send a recruitment pack and schedule a baseline testing session. Participants will continue to be screened for the trial until 240 participants have been randomised.

5.2 Informed Consent

All patients must willingly consent after reading the participant information letter and being informed of the procedures to be followed, potential benefits, side effects, risks, and discomforts. Informed consent is required

before any study-specific procedure is performed.

5.3 Randomisation

Subjects will be randomly allocated in a ratio of 1:1:1 to the three study arms, subject to maintaining approximate balance regarding stratification. A research methods consultant with no patient contact will be responsible for randomisation which will be performed in a Statistical Analysis System using the Pocock-Simon minimisation algorithm.

5.4 Stratification

Patients will be stratified by: 1) age (<60 years≥); 2) current sexual activity level (no/minor-moderate) as assessed by QLQ-PR25 sexual activity score[88]; 3) previous prostatectomy (yes/no); 4) previous radiotherapy (yes/no); and 5) previous/current AST (yes/no).

6.0 CLINICAL PROCEDURES

6.1 Guidelines for Pre-Study Testing:

To be completed before randomisation:

- Demographics Questionnaire
- Informed Consent
- Medical Doctor Consent

Table 1. Summary of assessments.

	Screening	Intervention phase		Post-intervention phase	
	Recruitment (prior to baseline)	Baseline	Post intervention	6 month follow up	1 year follow up
Complete Clinical Screening Sheet	X				
Complete Patient Screening Sheet	X				
Post Recruitment Pack	X				
Informed Consent		X			
Medical Doctor Consent		X			
Demographics Questionnaire		X			
Randomisation		X			
**Laboratory Studies**					
Testosterone, CRP, PSA		X	X		X
**Body measurements**					
Height		X	X		X
Weight		X	X		X
DXA (whole body, hip, lumbar spine)		X	X		X
Resting blood pressure and heart rate		X	X		X
**Exercise**					

6m walk tests (normal, fast, backwards)		X	X		X
400m walk test		X	X		X
Repeated chair rise test		X	X		X
1 RM (chest press, leg press)		X	X		X
**Safety**					
Adverse Events		X	X	X	X
**Hand outs**					
Questionnaires		X	X	X	X
Activity monitor and instructions		X	X		X
Activity Log		X	X		X

6.2 Measurements

All measurement study endpoints will take place at baseline, post-intervention (6 months) and 1-year follow-up (month 18). Self-report measures will also be assessed at 6 months follow-up (month 12). All assessment tools/procedures have established validity and reliability and are used widely in clinical research including by our team.

6.3 Primary Study Endpoint

6.3.1 Sexual Health

The IIEF will be utilised to assess sexual health across a variety of domains including erectile function, orgasmic function, sexual desire, intercourse satisfaction and overall satisfaction[86]. The Sexual Function scale of the EPIC is a prostate cancer specific tool which will be utilised to assess sexual function and satisfaction[87]. Sexual activity level will be assessed using the sexual domain of the European Organisation for Research and Treatment of Cancer (EORTC) prostate cancer specific questionnaire (QLQ-PR25)[88].

6.4 Secondary Study Endpoints

6.4.1 Sexual Self-Confidence

The Short Form Psychological and Interpersonal Relationship Scale will be used to assess sexual self-confidence[89].

6.4.2 Masculine Self-Esteem

The Masculine Self-Esteem Scale is designed specifically for use in men with prostate cancer and will be utilised to assess participant's appraisal of their masculinity[90].

6.4.3 Utilisation of Sexual Aids

A previously developed scale will assess whether participants have sought medical assistance for sexual dysfunction at any point before, during or after prostate cancer treatment[33]. All treatments will be documented and the impact of these treatments rated.

6.4.4 Relationship Satisfaction

The Dyadic Adjustment Scale will assess relationship satisfaction between participants and their partners[91].

6.4.5 Sexual Supportive Care Needs

The level of need for help with sexual dysfunction will be assessed by the sexuality domain of the Supportive Care Needs Survey[92].

6.4.6 Quality of Life

Health-related quality of life will be assessed using the Medical Outcomes Short Form 36 (SF-36)[93]. Quality of life will also be assessed using a prostate cancer specific tool, the EORTC QLQ-C30 and QLQ-PR25

questionnaires[94].

6.4.7 Urinary, Bowel & Hormonal Issues

Adverse side effects specific to prostate cancer treatments will be assessed using the urinary function, bowel habits and hormonal function scales of the EPIC[90].

6.4.8 Psychological Distress

The Brief Symptom Inventory-18 will be utilised to assess psychological distress across the following domains: anxiety, depression, somatisation and global distress severity[95]. Antidepressant use will also be recorded.

6.4.9 Fatigue

Cancer related fatigue will be assessed using the Functional Assessment of Chronic Illness Therapy-Fatigue questionnaire[96].

6.4.10 Body Composition

Regional and whole body lean mass and fat mass will be derived from whole body dual-energy X-ray absorptiometry scans. Trunk adiposity, visceral fat and adipose indices will be assessed using standard procedures.

6.4.11 Body Image

The Body Image Scale is a cancer-specific scale that will be used to assess participants perceptions of their appearance[97].

6.4.12 Physical Function & Physical Activity Levels

A series of standard tests will be used to assess physical function: 1) 400-m walk (aerobic capacity), 2) one repetition maximum in the leg press and chest press (muscular strength), 3) repeated chair rise (muscular power), 4) usual and fast pace 6-m walk (ambulation), and 5) backwards tandem 6-m walk (balance). Physical activity levels will be assessed objectively over a 7-day period using a validated, reliable tri-axial accelerometer activity monitor (ActiGraph GT3X+)[98]. Self-reported physical activity will also be assessed by the leisure score index from the Godin Leisure-Time Exercise Questionnaire[99].

6.4.13 Blood Biomarkers

Blood samples will be collected and analysed commercially by accredited Australian National Association of Testing Authorities laboratories for testosterone, high sensitivity C-reactive protein (CVD risk; also related to severity of penile vascular disease) and PSA.

Please see Appendix 1 for testing procedures.

6.5 Study Assessments by Visit

6.5.1 Baseline Assessment

6.5.1.1 Record date of visit

6.5.1.2 Collect informed consent, GP consent and demographic questionnaire.

6.5.1.3 Confirm blood sample was collected

6.5.1.4 Confirm baseline questionnaires were completed

6.5.1.5 Record any relevant medical history

6.5.1.6 Record anthropomorphic measurements (height, weight)

6.5.1.7 Perform DXA scans (whole body; hip; lumbar spine)

6.5.1.8 Record resting blood pressure and heart rate

6.5.1.9 Perform 6m walk tests (normal; fast; backwards)

6.5.1.10 Perform 400m walk test

6.5.1.11 Perform chair rise test

6.5.1.12 Complete 1 repetition maximum (RM) testing; chest press and leg press

6.5.1.13 Provide adverse event form

6.5.1.14 Provide guidelines for self-directed exercise and activity log (usual care only)

6.5.2 Post Intervention Assessment

6.5.2.1 Record date of visit

6.5.2.2 Confirm blood sample was collected

6.5.2.3 Confirm post intervention questionnaires were completed

6.5.2.4 Record any relevant medical history

6.5.2.5 Record anthropomorphic measurements (height, weight)

6.5.2.6 Perform DXA scans (whole body; hip; lumbar spine)

6.5.2.7 Record resting blood pressure and heart rate

6.5.2.8 Perform 6m walk tests (normal; fast; backwards)

6.5.2.9 Perform 400m walk test

6.5.2.10 Perform chair rise test

6.5.2.11 Complete 1 repetition maximum (RM) testing; chest press and leg press

6.5.2.12 Provide adverse event form

6.5.3 6 Month Follow Up Assessment

6.5.3.1 Confirm 6 month questionnaires were completed

6.5.3.2 Provide adverse event form

6.5.4 1 Year Follow Up Assessment

6.5.4.1 Record date of visit

6.5.4.2 Confirm blood sample was collected

6.5.4.3 Confirm post intervention questionnaires were completed

6.5.4.4 Record any relevant medical history

6.5.4.5 Record anthropomorphic measurements (height, weight)

6.5.4.6 Perform DXA scans (whole body; hip; lumbar spine)

6.5.4.7 Record resting blood pressure and heart rate

6.5.4.8 Perform 6m walk tests (normal; fast; backwards)

6.5.4.9 Perform 400m walk test

6.5.4.10 Perform chair rise test

6.5.4.11 Complete 1 repetition maximum (RM) testing; chest press and leg press

6.5.4.12 Provide adverse event form

6.5.5 Exercise session (3 times per 7 days)

Please Refer to Appendix 2 (Periodised Exercise Program)

7.0 EXERCISE INTERVENTION

The exercise intervention involves a combination of aerobic and resistance exercise performed during 3 sessions per week for 6 months. The program will be supervised by accredited exercise physiologists in various exercise clinics in Perth (5 sites), Brisbane, NSW Central Coast and NSW North Coast. The exercise sessions will be conducted in small groups of up to 10-12 participants exercising in pairs or under direct supervision to ensure correct technique and minimise the risk for injury. The exercise program is designed to provide optimal stimulus to the cardiorespiratory and neuromuscular systems while maximising safety, compliance and retention.

7.1 Resistance Training

Resistance exercise will involve 6-8 exercises that target the major upper and lower body muscle groups. Intensity will be manipulated from 6-12 repetition maximum (RM; i.e. the maximal weight that can be lifted 6 to 12 times which is equivalent to ~60-85% of 1RM) using 1-4 sets per exercise. To ensure the progressive nature of the training program, participants will be encouraged to work past the specific RM prescribed. The resistance will be increased by a 5-10% increment for the next set/training session if the subject is able to perform more repetitions than the RM specified during a set. Example exercises are below.

Lower Body
Leg press
Leg Curl
Leg Extension
Calf Raise
Lunges

Upper Body
Chest Press
Seated Row
Lat Pulldown
Biceps Curl
Tricep Extension
Shoulder Press

7.2 Aerobic Training

The aerobic exercise component will include 20 to 30 minutes of moderate to vigorous intensity cardiovascular exercise (~60-85% of estimated maximum heart rate) using a variety of modes such as walking or jogging on a treadmill, cycling or rowing on a stationary ergometer. Participants will be encouraged to undertake additional aerobic exercise outside the clinic sessions with the goal of achieving a total of at least 150 minutes of moderate to vigorous intensity aerobic exercise each week. Exercise prescription will be progressive and modified according to individual response. Both continuous and high intensity interval training will be implemented to provide greater variety and training stimulus. In order to reduce the possibility of boredom and overreaching, the exercise program will be periodised by cycling emphasis on intensity and volume. The exercise intervention has been designed in accordance with national[64] and international guidelines[42]. Furthermore, we have used this exercise prescription effectively in previous trials involving men with prostate cancer and have reported significant improvements in quality of life, lean muscle mass, fatigue, aerobic capacity, muscular strength, physical function and C-reactive protein[63,43,57,69].

7.3 Missed sessions

The number of missed sessions per participant and the reasons for missing sessions will be recorded and reported. If a participant misses exercise sessions they will be contacted by the research team to determine the reason.

7.4 Return to Exercise Training Following an Adverse Event

Return to exercise following reporting of an adverse event which requires updated physician clearance will be discussed on a case-by-case basis with the study coordinator. Where physician clearance to return to

exercise is deemed necessary, the participant will only be allowed to return to exercise training when physician clearance is provided in writing. The exercise physiologist supervising the exercise program will modify the program accordingly at re-introduction. If no physician clearance is deemed necessary, the participant can return to training upon clearance from the exercise physiologist.

7.5 Safety and Monitoring

Patients will be recruited in Perth WA, Brisbane QLD, Central Coast NSW and North Coast NSW by invitation of their attending specialist (radiation oncologist/urologist), with clinicians overseeing all aspects of management through our established team.

8.0 EXERCISE AND PSYCHOSEXUAL INTERVENTION

Participants receiving the combined exercise and psychosexual intervention will complete the exercise intervention described above as well as a brief psychosexual self-management intervention that addresses psychological and sexual well-being. A low intensity psycho-logical care approach will be utilised in order to maximise outcomes and accessibility (i.e. facilitates translation)[100]. Specifically, the intervention involves a single psycho-educational session and a self-management kit. At baseline, participants will receive a 60-minute face to face psycho-educational session with their exercise physiologist that addresses: stress management; problem solving coping for treatment challenges; and goal setting for sexual rehabilitation. These sessions will be audiotaped with 15% reviewed to ensure adherence to the intervention protocol. The psychosexual intervention will apply cognitive behavioral strategies; will utilise an adult learning approach in which men self-select goals to focus on; and will encourage self-management[101]. To support self-management men will receive a psychosexual kit that includes a published self-help book for men with prostate cancer and their partners[102]; study specific tip sheets about treatments for erectile dysfunction and goal setting for sexual rehabilitation; a progress journal-diary; and audio resources for stress management. The intervention delivered by the exercise physiologist will be manualised and based on existing materials already developed and trialled by the team[70,83]. The exercise physiologists will receive extensive training in how to deliver the intervention and continued supervision. Treatment fidelity will be managed consistent with NIH guidelines[103]. This pragmatic approach has been adopted based on the fact that men are low help-seekers and incorporating strategies that are linked to masculine ideals (i.e. peer support offered through exercising in a group/'team' of men rather than a traditional support group setting) may be more accepted by men and effective[104-106,pilot work].

9.0 USUAL CARE

The usual care group will be offered participation in the interventions at the completion of the 6-month period (half to receive the Exercise Intervention and half the Exercise + Psychosexual Intervention).

10.0 STATISTICAL CONSIDERATIONS

10.1 Calculation of Sample Size

Data from our 3-month study in prostate cancer patients indicates that the standard deviation for change in our primary outcome of sexual health equates to ~22 points. We observed the mean change after the intervention to differ between exercise and usual care groups by ~11 points. There is no available evidence comparing changes in sexual health between exercise and psychosexual interventions. However, data from previous psychosexual interventions in prostate cancer patients indicates changes of a moderate standardised effect (d = 0.5) in sexual health following the psycho-educational interventions[21,22]. We have based sample size calculations on the assumption that there will be an additive effect of exercise and psychosexual therapies. A priori, 64 participants per group will be required to achieve 80% power at an alpha level of 0.05 (two tailed), and to demonstrate a difference between the three groups in sexual health at the end of the intervention. Previous experience in exercise and psychosexual therapy trials indicates an attrition rate of up to 20% over the intervention period. Therefore, to adequately ensure that we have sufficient participant numbers at the end of the intervention, 240 participants will be randomised to the study arms. A sample size of 240 will also provide us with sufficient power to detect a moderate standardised effect (d = 0.5) in our secondary outcomes. Based on previous experience we anticipate a further attrition rate of up to 20% over the follow-up period. Therefore, the sample size will provide 87% statistical power to detect differences between the exercise group

and the exercise + psychosexual group at follow-up (d = 0.5; n = 77 per group as the usual care group will be split between the two interventions for the prospective cohort study component of this project).

10.2 Statistical Analysis

Analyses will be conducted using IBM SPSS Statistics (latest version) (IBM corp., Armonk, NY) with an intention-to-treat approach using maximum likelihood imputation of missing values (expectation maximization). Normality of the distribution will be assessed using the Kolmogorov-Smirnov test. Analyses will include standard descriptive statistics, chi-square, correlation and regression, and analysis of variance (ANOVA) or covariance (ANCOVA). Differences at baseline will be assessed using one-way analysis of variance (ANOVA) or the Kruskal-Wallis test, as appropriate, for continuous data and Chi-square for categorical data. For the primary and secondary outcomes, a two-way (group x time) repeated-measures ANOVA or ANCOVA, as appropriate, will be undertaken. Follow-up tests will be performed if the interaction or the main effect of time is significant. Data not normally distributed will be log transformed (ln) for analysis. Initially the exercise and exercise plus self-managed psychosexual therapy will be combined and compared to usual care. If exercise and exercise plus self-managed psychosexual therapy are effective for improving sexual health, then the additional effects of exercise and self-managed psychosexual therapy will be compared to exercise alone by using a two-way (group x time) repeated-measures ANOVA (or ANCOVA as appropriate). To examine the effect of the intervention based on baseline sexual function, trend analysis will be performed using linear regression and entering tertiles of the sexual health domains as an ordinal variable. All tests will be two-tailed with an alpha level of 0.05 for statistical significance.

11.0 ADVERSE EVENTS AND REPORTING REQUIREMENTS

Adverse events (AEs) will be assessed at every clinic visit and the following details recorded on the AE record form: type, incidence, severity, timing, seriousness, and relatedness to a) disease and b) the intervention.

11.1 Definitions

11.1.1 Adverse Events (AE)

An adverse event is any unfavorable or unintended sign, symptom, or disease temporally associated with the study intervention and occurring in a patient assigned to the intervention. All AEs will be considered. This includes adverse clinical or laboratory findings, illness, or an exacerbation or progression of a disease/condition present at baseline.

Rare but serious AEs are possible. Those that may occur during either exercise testing or exercise training include myocardial infarction (heart attack), stroke, unconsciousness or other serious injury.

11.1.2 Serious Adverse Events (SAE)

A serious adverse event (SAE) is any adverse event occurring at any level of intervention that results in any of the following outcomes:

- Results in death. *If the malignancy under study has a fatal outcome during the study or within the safety reporting period, the event leading to death should be reported as a Grade 5 SAE; death is an outcome and not the adverse event in itself.*
- Is life-threatening (i.e., immediate risk of death from the reaction as it occurred). *It does **not** include a reaction which hypothetically might have caused death had it occurred in a more severe form.*
- Requires or prolongs inpatient hospitalization (i.e., the event required at least a 24-hour hospitalization or prolonged a hospitalization beyond the expected length of stay). Hospitalization admissions and/or surgical operations scheduled to occur during the study period, but planned prior to study entry are not considered SAEs if the illness or disease existed before the person was enrolled in the trial, provided that it did not deteriorate in an unexpected manner during the trial (e.g., surgery performed earlier than planned)
- Results in persistent or significant disability/incapacity. Disability is defined as a substantial disruption of a person’s ability to conduct normal life functions

- Is an important medical event when, based upon appropriate medical judgment, it may jeopardize the participant and require medical or surgical intervention to prevent one of the outcomes listed above.

Events **not** considered to be SAEs are hospitalizations for:

- Routine treatment or monitoring of the studied indication, not associated with any deterioration in condition
- Elective or pre-planned treatment for a pre-existing condition that did not worsen
- Emergency outpatient treatment for an event not fulfilling the serious criteria outlined above and not resulting in inpatient admission

11.1.3 Non-Serious Adverse Events

All other events.

11.2 Attribution

A suspected adverse reaction means any adverse event for which there is reasonable possibility that the intervention caused the adverse event. For the purposes of safety reporting, "reasonable possibility" means there is evidence to suggest a causal relationship between the intervention and the adverse event.

The Investigator will assign attribution of the possible association of the event with the study intervention using the following definitions:

Unrelated to the exercise intervention: The adverse event is *clearly not related* or is *doubtfully related* to the exercise intervention

Related to exercise intervention: The adverse event *may be related,* is *likely related,* or is *clearly related* to the exercise intervention

11.3 Reporting Requirements

All AEs and SAEs whether reported by the patient, discovered during questioning, directly observed, or detected by physical examination, laboratory test or other means must be recorded in the patient's AE record and on the appropriate AE form.

11.3.1 SAE Reporting

All SAEs occurring during the study must be reported to the appropriate Study contact person by study-site personnel within 24 hours of their knowledge of the event. This timeframe also applies to additional new information (follow-up). SAEs should be reported by facsimile or email. The names (and corresponding telephone numbers) of the individuals who should be contacted regarding safety issues or questions regarding the study will be provided as a separate document. The Investigator is also responsible for notifying the Human Research Ethics Committee (HREC) in accordance with local regulations. All events should be followed to their resolution, until the Investigator assesses them as stable, irreversible, or until the patient is lost to follow-up, whichever comes first.

11.3.2 Non-Serious AE Reporting

Adverse events should be recorded on the AE form from the time the patient has signed the informed consent at baseline until completion of the 1-year follow-up assessment. All events should be followed to their resolution, until the Investigator assesses them as stable, irreversible, or until the patient is lost to follow-up, whichever comes first.

11.4 AE Monitoring

The Investigators and Study Coordinators will monitor AEs throughout the study period to review safety procedures and protocols.

12.0 WITHDRAWAL OF PARTICIPANTS

12.1 Withdrawal of participants

Participants are free to withdraw from the trial at any stage without providing a reason and without consequence. This information will be stated in the participant information letter. Participants can inform the research team at their local site of their decision to withdraw. If a participant withdraws from the study, any data collected on him up to that point in the study will go forward for study analysis. This information will be stated in the participant information letter.

13.0 DATA MANAGEMENT AND MONITORING

At the completion of every data collection session, any physical documentation should be stored in a secure, locked filing cabinet, accessible only by study staff. The exercise program logs for patients attending exercise clinics will be stored within a locked, secure locker. Electronic data collected should be stored on password protected computers accessible only study staff.

14.0 AMENDMENTS

July 2015: This study is being amended to allow inclusion of participants who are <12 months since prostatectomy or completion of radiotherapy or AST.

15.0 REFERENCES

1. 1. Cormie P, et al: Exercise maintains sexual activity in men undergoing androgen suppression for prostate cancer: a randomized controlled trial. Prostate Cancer Prostatic Dis 16:170-175, 2013
2. Canada AL, et al: Pilot intervention to enhance sexual rehabilitation for couples after treatment for localized prostate carcinoma. Cancer 104:2689-2700, 2005
3. Schover LR, et al: A randomized trial of internet-based versus traditional sexual counseling for couples after localized prostate cancer treatment. Cancer 118:500-509, 2012
4. Reese JB: Coping with sexual concerns after cancer. Curr Opin Oncol 23:313-321, 2011
5. Ottenbacher A, et al: Cancer-Specific Concerns and Physical Activity Among Recently Diagnosed Breast and Prostate Cancer Survivors. Integr Cancer Ther, 2012
6. Sanda MG, et al: Quality of life and satisfaction with outcome among prostate-cancer survivors. N Engl J Med 358:1250-61, 2008
7. Bober SL, et al: Sexuality in Adult Cancer Survivors: Challenges and Intervention. J Clin Oncol 30:3712-3719, 2012
8. Sadovsky R, et al: Cancer and sexual problems. J Sex Med 7:349-73, 2010
9. Resnick MJ, et al: Long-term functional outcomes after treatment for localized prostate cancer. N Engl J Med 368:436-45, 2013
10. Stanford JL, et al: Urinary and sexual function after radical prostatectomy for clinically localized prostate cancer: the Prostate Cancer Outcomes Study. JAMA 283:354-60, 2000
11. Spry NA, et al: Adverse effects to quality of life arising from treatment can recover with intermittent androgen suppression in men with prostate cancer. Eur J Cancer 42:1083-92, 2006
12. Hamilton AS, et al: Health outcomes after external-beam radiation therapy for clinically localized prostate cancer: results from the Prostate Cancer Outcomes Study. J Clin Oncol 19:2517-26, 2001
13. Parker WR, et al: Five year expanded prostate cancer index composite-based quality of life outcomes after prostatectomy for localized prostate cancer. BJU Int 107:585-90, 2011
14. Higano CS: Sexuality and Intimacy After Definitive Treatment and Subsequent Androgen Deprivation Therapy for Prostate Cancer. J Clin Oncol 30:3720-3725, 2012
15. Bacon CG, et al: Sexual function in men older than 50 years of age: results from the health professionals

follow-up study. Ann Intern Med 139:161-8, 2003

16. Penson DF, et al: Is Quality if Life Different for Men with Erectile Dysfunction and Prostate Cancer Compared to Men with Erectile Dysfunction Due to Other Causes? Results from the ExCEED Data Base. J Urol 169:1458-1461, 2003

17. Elliott S, et al: Androgen Deprivation Therapy for Prostate Cancer: Recommendations to Improve Patient and Partner Quality of Life. J Sex Med 7:2996-3010, 2010

18. Seftel AD: Erectile Dysfunction in the Elderly: Epidemiology, Etiology and Approaches to Treatment. J Urol 169:1999-2007, 2003

19. Derby CA, et al: Modifiable risk factors and erectile dysfunction: can lifestyle changes modify risk? Urology 56:302-6, 2000

20. Bacon CG, et al: A Prospective Study of Risk Factors for Erectile Dysfunction. J Urol 176:217-221, 2006

21. Sharifi N, et al: Androgen deprivation therapy for prostate cancer. JAMA 294:238-44, 2005

22. Galvao DA, et al: Exercise can prevent and even reverse adverse effects of androgen suppression treatment in men with prostate cancer. Prostate Cancer Prostatic Dis 10:340-6, 2007

23. Collier A, et al: Prostate cancer, androgen deprivation therapy, obesity, the metabolic syndrome, type 2 diabetes, and cardiovascular disease: a review. Am J Clin Oncol 35:504-9, 2012

24. Levine GN, et al: Androgen-deprivation therapy in prostate cancer and cardiovascular risk: a science advisory from the American Heart Association, American Cancer Society, and American Urological Association: endorsed by the American Society for Radiation Oncology. Circulation 121:833-40, 2010

25. Zaider T, et al: Loss of Masculine Identity, Marital Affection, and Sexual Bother in Men with Localized Prostate Cancer. J Sex Med 9:2724-2732, 2012

26. Klaeson K, et al: Sexuality in the context of prostate cancer narratives. Qual Health Res 22:1184-94, 2012

27. Hoyt MA, et al: Cancer-related masculine threat, emotional approach coping, and physical functioning following treatment for prostate cancer. Health Psychol 32:66-74, 2013

28. Miles CL, et al: Interventions for sexual dysfunction following treatments for cancer. Cochrane Database Syst Rev:CD005540, 2007

29. Magheli A, et al: Erectile dysfunction following prostatectomy: prevention and treatment. Nat Rev Urol 6:415-427, 2009

30. Peltier A, et al: Current management of erectile dysfunction after cancer treatment. Curr Opin Oncol 21:303-309, 2009

31. Burnett AL: Erectile Dysfunction. J Urol 175:S25-S31, 2006

32. Wespes E, et al: EAU Guidelines on Erectile Dysfunction: An Update. Eur Urol 49:806-815, 2006

33. Schover LR, et al: The use of treatments for erectile dysfunction among survivors of prostate carcinoma. Cancer 95:2397-407, 2002

34. Stephenson RA, et al: Treatment of erectile dysfunction following therapy for clinically localized prostate cancer: patient reported use and outcomes from the Surveillance, Epidemiology, and End Results Prostate Cancer Outcomes Study. J Urol 174:646-50; discussion 650, 2005

35. Tal R, et al: Penile implant utilization following treatment for prostate cancer: analysis of the SEER-Medicare database. J Sex Med 8:1797-804, 2011

36. Nelson CJ, et al: Sexual bother following radical prostatectomy. J Sex Med 7:129-35, 2010

37. Brotto LA, et al: Psychological interventions for the sexual sequelae of cancer: a review of the literature. J Cancer Surviv 4:346-60, 2010

38. Melnik T, et al: Psychosocial interventions for erectile dysfunction. Cochrane Database Syst

Rev:CD004825, 2007

39. Faller H, et al: Effects of Psycho-Oncologic Interventions on Emotional Distress and Quality of Life in Adult Patients With Cancer: Systematic Review and Meta-Analysis. J Clin Oncol 31:782-793, 2013

40. Dahn JR, et al: Physical activity and sexual functioning after radiotherapy for prostate cancer: beneficial effects for patients undergoing external beam radiotherapy. Urology 65:953-8, 2005

41. Segal RJ, et al: Randomized controlled trial of resistance or aerobic exercise in men receiving radiation therapy for prostate cancer. J Clin Oncol 27:344-51, 2009

42. Schmitz K, et al: American College of Sports Medicine Roundtable on Exercise Guidelines for Cancer Survivors. Med Sci Sports Exerc 42:1409-1426, 2010

43. Galvao DA, et al: Combined resistance and aerobic exercise program reverses muscle loss in men undergoing androgen suppression therapy for prostate cancer without bone metastases: a randomized controlled trial. J Clin Oncol 28:340-347, 2010

44. Galvão DA, et al: Physical Activity and Cancer; Series: Recent Results Cancer Research Physical Activity and Genitourinary Cancer Survivorship 186: 217-236, 2011

45. Nicolosi A, et al: Epidemiology of erectile dysfunction in four countries: cross-national study of the prevalence and correlates of erectile dysfunction. Urology 61:201-6, 2003

46. Pinnock CB, et al: Erectile dysfunction in the community: a prevalence study. Med J Aust 171:353-7, 1999

47. Hsiao W, et al: Exercise is associated with better erectile function in men under 40 as evaluated by the International Index of Erectile Function. J Sex Med 9:524-30, 2012

48. White JR, et al: Enhanced sexual behavior in exercising men. Arch Sex Behav 19:193-209, 1990

49. Esposito K, et al: Effect of lifestyle changes on erectile dysfunction in obese men: a randomized controlled trial. JAMA 291:2978-84, 2004

50. Esposito K, et al: Effects of Intensive Lifestyle Changes on Erectile Dysfunction in Men. J Sex Med 6:243-250, 2009

51. Spry NA, et al: Long-term effects of intermittent androgen suppression therapy on lean and fat mass: a 33-month prospective study. Prostate Cancer and Prostatic Dis 16:67-72, 2013

52. Galvao DA, et al: Reduced muscle strength and functional performance in men with prostate cancer undergoing androgen suppression: a comprehensive cross-sectional investigation. Prostate Cancer and Prostatic Dis 12:198-203, 2009

53. Galvao DA, et al: Changes in muscle, fat and bone mass after 36 weeks of maximal androgen blockade for prostate cancer. BJU Int 102:44-47, 2008

54. Galvao DA, et al: Cardiovascular and metabolic complications during androgen deprivation: exercise as a potential countermeasure. Prostate Cancer and Prostatic Dis 12:233-240, 2009

55. Dunn J, et al: Trajectories of Psychological Distress after Colorectal Cancer Psychoonocology 22:1759-1765, 2013

56. Chambers SK, et al: Mindfulness Groups for Men with Advanced Prostate Cancer: Feasibility, Effectiveness and the Role of Peer Support. Support Care Cancer 20:1183-1192, 2012

57. Newton RU, et al: A phase III clinical trial of exercise modalities on treatment side-effects in men receiving therapy for prostate cancer. BMC Cancer 9:210, 2009

58. Galvao DA, et al: Review of exercise intervention studies in cancer patients. J Clin Oncol 23:899-909, 2005

59. Galvao DA, et al: Resistance training and reduction of treatment side effects in prostate cancer patients. Med Sci Sports Exerc 38:2045-52, 2006

60. Newton RU, et al: Guidelines for implementing exercise programs for cancer patients. Perth, WA, Cancer Council of Western Australia, 2007 pp. 114

61. Bolam K, et al: AST-induced Bone Loss in Men with Prostate Cancer: Exercise as a Potential Countermeasure. Prostate Cancer and Prostatic Dis 15:329-338, 2012

62. Newton RU, et al: Can exercise ameliorate treatment toxicity during the initial phase of testosterone deprivation in prostate cancer patients? Is this more effective than delayed rehabilitation? BMC Cancer 12:432, 2012

63. Galvao DA, et al: Acute Versus Chronic Exposure to Androgen Suppression for Prostate Cancer: Impact on the Exercise Response. J Urol 186:291-1297, 2011

64. Hayes SC, et al: Australian Association for Exercise and Sport Science position stand: Optimising cancer outcomes through exercise. J Sci Med Sport 12:428-434, 2009

65. Galvao DA, et al: Endocrine and immune responses to resistance training in prostate cancer patients. Prostate Cancer and Prostatic Disease (2008) 11 2 160-165

66. Galvao DA, et al: Cardiovascular and metabolic complications during androgen deprivation: exercise as a potential countermeasure. Prostate Cancer and Prostatic Dis 12:233-240, 2008

67. Newton RU, et al: Exercise in prevention and management of cancer. Curr Treat Options Oncol, 9:135-146, 2008

68. Galvão DA, et al: Efficacy and safety of a modular multi-modal exercise program in prostate cancer patients with bone metastases: a randomized controlled trial. BMC Cancer 11:517, 2011

69. Galvao DA, et al: A randomized controlled trial of an exercise intervention targeting cardiovascular and metabolic risk factors for prostate cancer patients from the RADAR trial. BMC Cancer 9:419, 2009

70. Lose F, et al: Genetic Variation in the KLK4 Locus is Associated with Risk of Prostate Cancer PLoS One 7:e44520, 2012

71. Chambers SK, et al: Mindfulness Groups for Men with Advanced Prostate Cancer: Feasibility, Effectiveness and the Role of Peer Support. Support Care Cancer 26:1183-1192, 2012

72. Chambers SK, et al: Survivor identity after colorectal cancer: Antecedents, prevalence and outcomes. Psychooncology 21:962-969, 2012

73. Ownsworth T, et al: Applying a biopsychosocial perspective to investigate factors related to emotional adjustment and quality of life for individuals with brain tumour. Brain Impairment 11:270-280, 2010

74. Hawkes A, et al: Feasibility of psychological distress screening by a community-based Cancer Helpline: Use the distress thermometer. BMC Cancer 14:14, 2010

75. McDowell ME, et al: Predictors of Change in Unmet Supportive Care Needs in Cancer. Psychooncology 19:508-516, 2010

76. McDowell M, et al: Prospective Predictors of Psychosocial Support Service Use after Cancer. Psychooncology 20:788-791, 2010

77. Chambers SK, et al: Living with prostate cancer: randomised controlled trial of a multimodal supportive care intervention for men with prostate cancer. BMC Cancer 11:317, 2011

78. Baade PD, et al: When do I know I am cured? Using conditional estimates to provide better information about survival prospects. Med J Aust 194:73-77, 2011

79. Chambers SK, et al: A Systematic Review of Psychosocial Interventions for Men with Prostate Cancer and Their Partners. Patient Educ Couns 85:e75-88, 2011

80. Chambers SK, et al: ProsCan for Couples: A Feasibility Study for Evaluating Peer Support within a Controlled Research Design Psychooncology 22:475-479, 2013

81. Lose F, et al: The Kallikrein 14 is downregulated by androgen receptor signalling and harbours genetic

variation that is associated with prostate tumour aggressiveness. Biol Chem 393:403-412, 2012

82. Chambers SK, et al: Beating the Blues after Cancer: Randomised controlled tele-based psychological intervention for high distress patients and carers. BMC Cancer 9:189, 2009

83. Chambers SK, et al: ProsCan for Couples: Randomised controlled trial of a couples-based sexuality intervention for men with localised prostate cancer who receive radical prostatectomy. BMC Cancer 8:226, 2008

84. Chambers SK, et al: ProsCan for Men: Randomised controlled trial of a decision support intervention for men with localised prostate cancer. BMC Cancer 8:207, 2008

85. Schulz KF, et al: CONSORT 2010 Statement: updated guidelines for reporting parallel group randomised trials. BMC Med 8:18, 2010

86. Rosen RC, et al: The international index of erectile function (IIEF): a multidimensional scale for assessment of erectile dysfunction. Urology 49:822-30, 1997

87. Wei JT, et al: Development and validation of the expanded prostate cancer index composite (EPIC) for comprehensive assessment of health-related quality of life in men with prostate cancer. Urology 56:899-905, 2000

88. van Andel G, et al: An international field study of the EORTC QLQ-PR25: a questionnaire for assessing the health-related quality of life of patients with prostate cancer. Eur J Cancer 44:2418-24, 2008

89. Swindle R, et al: A 15-item short form of the psychological and interpersonal relationship scales. Int J Impot Res 18:82-8, 2006

90. Clark JA, et al: Measuring patients' perceptions of the outcomes of treatment for early prostate cancer. Med Care 41:923-36, 2003

91. Sharpley C, et al: A psychometric evaluation of the Spanier Dyadic Adjustment Scale. J Marriage Fam 44:739-741, 1982

92. Bonevski B, et al: Evaluation of an instrument to assess the needs of patients with cancer. Supportive Care Review Group. Cancer 88:217-25, 2000

93. Ware JE, Jr., et al: The MOS 36-item short-form health survey (SF-36). I. Conceptual framework and item selection. Med Care 30:473-83, 1992

94. Aaronson NK, et al: The European Organization for Research and Treatment of Cancer QLQ-C30: a quality-of-life instrument for use in international clinical trials in oncology. J Natl Cancer Inst 85:365-76, 1993

95. Zabora J, et al: A new psychosocial screening instrument for use with cancer patients. Psychosomatics 42:241-6, 2001

96. Yellen SB, et al: Measuring fatigue and other anemia-related symptoms with the Functional Assessment of Cancer Therapy measurement system. J Pain Symptom Manage 13:63-74, 1997

97. Hopwood P, et al: A body image scale for use with cancer patients. Eur J Cancer 37:189-197, 2001

98. Plasqui G, et al: Physical activity assessment with accelerometers: an evaluation against doubly labeled water. Obesity (Silver Spring) 15:2371-9, 2007

99. Jacobs DR, Jr., et al: A simultaneous evaluation of 10 commonly used physical activity questionnaires. Med Sci Sports Exerc 25:81-91, 1993

100. Bennett-Levy J, et al: Low Intensity CBT Interventions: A Revolution in Mental Health Services, in Bennett-Levy J, et al (eds): Oxford Guide to Low Intensity CBT Interventions. Oxford, Oxford University Press, 2010, pp 3-18

101. Barlow J, et al: Self-management approaches for people with chronic conditions: a review. Patient Educ Couns 48:177-87, 2002

102. Chambers SK Facing the Tiger: A Guide for Men with Prostate Cancer and the People who Love Them (2012)

103. Bellg AJ, et al: Enhancing treatment fidelity in health behavior change studies: best practices and recommendations from the NIH Behavior Change Consortium. Health Psychol 23:443-51, 2004

104. Addis ME, et al: Men, masculinity, and the contexts of help seeking. Am Psychol 58:5-14, 2003

105. Galdas PM, et al: Men and health help-seeking behaviour: literature review. J Adv Nurs 49:616-23, 2005

106. Smith JA, et al: What do we know about men's help-seeking and health service use? Med J Aust 184:81-3, 2006.

16.0 APPENDICES

Appendix 1 – AEP Testing Procedures
Appendix 2 – Periodised Exercise Program

APPENDIX 1 - AEP TESTING PROCEDURES

1.0 RECRUITMENT PACK

The following documents should be posted to the participant to be completed prior to the baseline assessment. The participant should obtain consent from their medical doctor, undergo the blood test and complete all forms and questionnaires and bring them to the baseline assessment.

1.1 Information Letter and Informed Consent

Outlines all aspects of the study. Should be reviewed by the participant and signed prior to baseline testing.

1.2 Medical Doctor Consent Form

This form needs to be signed by the participant's medical doctor prior to baseline testing.

1.3 Demographic and Health History Information

The Demographic and Health History questionnaire is used to obtain demographic information (e.g., date of birth, marital status, education) and will be completed by the participant prior to baseline testing.

1.4 Pathology Collection Form

Participants need to have a blood sample collected to measure testosterone, high sensitivity C-reactive protein and PSA. The test should be done at an Australian National Association of Testing Authorities accredited laboratory.

2.0 TESTING ASSESSMENTS

2.1 Weight

Body weight will be measured using a digital scale. Participants will be measured barefoot wearing one layer of light clothing. Weight will be recorded in kilograms (kg), rounded to 2 decimal place.

2.2 Height

Height will be measured using a standard stadiometer height measurement. Participants will stand in front of the stadiometer, in bare feet, arms relaxed by their sides and head in the Frankfort plane (eyes in line with the upper part of the ears). The assessor will slide the rod downwards until it rests on the participant's head to read height. Height will be recorded in centimeters (cm), rounded to 1 decimal place.

2.3 DXA Scans (whole body; hip; lumbar spine)

Regional and whole body lean mass and fat mass will be derived from whole body dual-energy X-ray absorptiometry scans. Trunk adiposity, visceral fat and adipose indices will be assessed using standard procedures.

2.4 Resting Blood Pressure

Participant should lay supine on a bed for at least five minutes prior to measuring blood pressure. Three measures of blood pressure should be taken with 30 seconds rest between each.

2.5 6m Walk Tests

The start and finish lines should be marked 6 meters apart. Participants should start with their feet shoulder width apart and in line with the start line.

2.5.1 Normal Walk

Have the participant walk at a normal/everyday pace past the 6m mark. The time taken to complete the test will be recorded. Complete three trials of the test.

2.5.2 Fast Walk

Have the participant walk as fast as possible (without running) past the 6m mark. The time taken to complete

the test will be recorded. Complete three trials of the test.

2.5.3 Backwards Walk

Position the participant with heels on start line. Instruct participant to walk as fast as possible heel to toe past 6m mark. The time taken to complete the test will be recorded. Complete three trials of the test.

2.6 400m Walk Test

The 400m walk test is a self-paced, submaximal exercise test. The time taken to complete the 400m course correlates well with VO_2max and will provide a surrogate measure of aerobic fitness during the intervention. Participants will be required to move as fast as they can along a 20m course, marked by two cones, until they have completed 10 laps of the course (400m). The time taken to complete the test will be recorded. Heart rate will be measured pre-test, immediately post-test, 1-minute post-test and 2-minutes post-test using a heart rate monitor. At the end of the test participants will complete a lap of slow walking as part of active recovery.

2.7 Repeated Chair Rise Test

Follow the procedure below:

- Position chair up against wall.
- Position participant on chair with back against back rest and arms across chest.
- Feet are shoulder width apart on floor with knees positioned at 90^0.
- Instruct participant to stand and sit five times as fast as possible.
- Ensure participant stands fully upright (knees straight) before returning to chair with upper back touching back rest on each stand/sit attempt.
- Inform participant to be wary of their head hitting the wall.
- Instruct participant with “3, 2, 1 Go”. Start stop watch on “go”
- Count out each stand and sit attempt
- Stop the stopwatch once the participant returns their back to the backrest on the 5th attempt.
- Complete three trials with 1 minute rest between trials.

2.8 Strength Assessments

Strength assessments will comprise one repetition maximum chest press, leg press and seated row. The 1-RM is defined as the highest load that can be lifted through full range of movement at one time.

2.8.1 Chest Press

1RM chest press can be completed using a pin-loaded machine, a Smith or barbell press or dumbbell press. A flat bench is required for Smith machine, barbell or dumbbell press exercises.

2.8.2 Leg Press

1RM leg press can be completed using a horizontal leg press or an incline leg press.

2.8.3 1 Repetition Maximum Testing Protocol

The assessor will ensure the correct starting position of the participant depending on the equipment used and instruct the participant on the correct testing technique. Testing will begin with a warm-up consisting of 6 repetitions at approximately 60% of 1RM with 2 minutes rest followed by 3 repetitions at approximately 80% 1RM with 2 minutes rest. 1RM will be determined across a maximum of 5 trials with a rest period of 2 minutes between each trial. Assessors should ensure that their level of motivation is consistent between and within participants and time points. All attempts and final successful attempt at 1RM should be recorded. Method of assessing 1RM at baseline should be noted and repeated for subsequent measures, i.e. equipment used and modifications to testing position.

3.0 BLOOD MARKERS

Testosterone, high sensitivity C-reactive protein, and PSA tests must be completed prior to baseline testing (section 1.4) and at post intervention and 1 year follow up assessments.

4.0 QUESTIONNAIRES

Participants will complete questionnaires at all assessment time points (baseline, post intervention, 6 month follow up and 1 year follow up). Questionnaires to be completed will include the following.

4.1 International Index of Erectile Function Questionnaires

4.2 EPIC

4.3 EORTC QLQ-C30

4.4 EORTC – QLQ-PR25

4.5 Medical Assistance for Sexual Problems

4.6 Sexual Self-efficacy Scale for Erectile Functioning

4.7 Short Form Psychological & Interpersonal Relationship Scale

4.8 Masculinity in Chronic Disease Inventory

Assesses the extent to which men identify with six masculine values: strength, sexual importance. priority, family responsibilities, emotional self-reliance, optimistic capacity, and action approach.

4.9 Prostate Cancer-related Quality of Life Scales

4.10 Personal Attributes Questionnaire

4.11 Dyadic Adjustment Scale

4.12 Supportive Care Needs – Sexuality

4.13 SF-36

Assesses health related quality of life.

4.14 Brief Symptom Inventory-18 (BSI-18)

Assesses psychological distress across the following domains: anxiety, depression, somatisation and global distress severity.

4.15 Antidepressant and Counselling Questionnaire

Records antidepressant use and psychiatrist, psychologist and counsellor visits.

4.16 FACIT Fatigue Scale

4.17 Body Image Scale

4.18 Godin Leisure Time Physical Activity Questionnaire

Self-administered, four-item questionnaire designed to measure an individual’s leisure-time activity during a typical week. Participants are asked to consider the number of occasions they spend per week, of at least 15 minutes duration, in strenuous, moderate and mild exercise. A total leisure score (TLS) is calculated as the sum of weekly frequencies of strenuous, moderate and vigorous intensity activity by their corresponding MET values: [TLS = (9 METs x strenuous activity time) + (5 METs x moderate activity time) + (3 METs x light activity time)]. The questionnaire also asks participants to consider how often they engage in activity long enough to work up a sweat with respondents choosing from the options: “often”, “sometimes” or “never/rarely”. The Godin questionnaire can be completed in 5 minutes.

APPENDIX 2 - PERIODISED EXERCISE PROGRAM

NAME: ____________________________________ **DOB**: ____________________ **WEEKS 1 - 2**

**Session #:**	**1**		**2**		**3**		**4**		**5**		**6**	
**Day|Date:**												

**Warm-up:**						

RESISTANCE EXERCISE: Target = 2 sets x 12 repetition maximum

	Reps	Wt	Reps	Wt	Reps	Wt	Reps	Wt	Reps	Wt	Reps	Wt
Leg Press												
Chest Press												
Leg Curl												
Seated Row												
Leg Extension												
Biceps Curl												
Calf Raise												
Tricep Ext.												

AEROBIC EXERCISE: Target = 20-30 mins Constant Intensity

	Time	Level	Time	Level	Time	Level	Time	Level	Time	Level	Time	Level
**Treadmill**												
**Rower**												
**Cross-Trainer**												
**Bicycle**												

**Stretching:**						

**Session RPE**						
**# in group**						

Comments / Reasons for Missing Sessions:

NAME: ________________________________ **DOB**: ____________________ **WEEKS 3 - 4**

**Session #:**	**7**		**8**		**9**		**10**		**11**		**12**	
**Day|Date:**												

**Warm-up:**						

RESISTANCE EXERCISE: Target = 3 sets x 12 repetition maximum

	Reps	Wt	Reps	Wt	Reps	Wt	Reps	Wt	Reps	Wt	Reps	Wt
Leg Press												
Chest Press												
Leg Curl												
Seated Row												
Leg Extension												
Biceps Curl												
Calf Raise												
Tricep Ext.												

AEROBIC EXERCISE: Target = 20-30 mins Constant Intensity

	Time	Level	Time	Level	Time	Level	Time	Level	Time	Level	Time	Level
**Treadmill**												
**Rower**												
**Cross-Trainer**												
**Bicycle**												

**Stretching:**						

**Session RPE**						
**# in group**						

Comments / Reasons for Missing Sessions:

NAME: ________________________________ **DOB:** ____________________ **WEEKS 5 - 6**

**Session #:**	**13**		**14**		**15**		**16**		**17**		**18**	
**Day|Date:**												

**Warm-up:**						

RESISTANCE EXERCISE: Target = 3 sets x 12 repetition maximum

	Reps	Wt	Reps	Wt	Reps	Wt	Reps	Wt	Reps	Wt	Reps	Wt
Leg Press												
Chest Press												
Leg Curl												
Seated Row												
Leg Extension												
Biceps Curl												
Calf Raise												
Tricep Ext.												

AEROBIC EXERCISE: Target = 30 mins Constant Intensity

	Time	Level	Time	Level	Time	Level	Time	Level	Time	Level	Time	Level
**Treadmill**												
**Rower**												
**Cross-Trainer**												
**Bicycle**												

**Stretching:**						

**Session RPE**						
**# in group**						

Comments / Reasons for Missing Sessions:

NAME: ______________________________ **DOB:** ____________________ **WEEKS 7 - 8**

**Session #:**	**19**		**20**		**21**		**22**		**23**		**24**	
**Day|Date:**												

**Warm-up:**						

RESISTANCE EXERCISE: Target = 3 sets x 10 repetition maximum *increase weights*

	Reps	Wt	Reps	Wt	Reps	Wt	Reps	Wt	Reps	Wt	Reps	Wt
Leg Press												
Chest Press												
Leg Curl												
Seated Row												
Leg Extension												
Biceps Curl												
Calf Raise												
Tricep Ext.												

AEROBIC EXERCISE: Target = 30 mins Constant Intensity

	Time	Level	Time	Level	Time	Level	Time	Level	Time	Level	Time	Level
**Treadmill**												
**Rower**												
**Cross-Trainer**												
**Bicycle**												

**Stretching:**						

**Session RPE**						
**# in group**						

Comments / Reasons for Missing Sessions:

NAME: ___________________________________ **DOB:** ____________________ **WEEKS 9 - 10**

**Session #:**	**25**		**26**		**27**		**28**		**29**		**30**	
**Day|Date:**												

**Warm-up:**						

RESISTANCE EXERCISE: Target = 3-4 sets x 10 repetition maximum

	Reps	Wt	Reps	Wt	Reps	Wt	Reps	Wt	Reps	Wt	Reps	Wt
Leg Press												
Chest Press												
Leg Curl												
Seated Row												
Leg Extension												
Biceps Curl												
Calf Raise												
Tricep Ext.												

AEROBIC EXERCISE: Target = 30 mins Constant Intensity

	Time	Level	Time	Level	Time	Level	Time	Level	Time	Level	Time	Level
**Treadmill**												
**Rower**												
**Cross-Trainer**												
**Bicycle**												

**Stretching:**						

**Session RPE**						
**# in group**						

Comments / Reasons for Missing Sessions:

NAME: ___________________________________ **DOB**: ____________________ **WEEKS 11 - 12**

**Session #:**	**31**		**32**		**33**		**34**		**35**		**36**	
**Day|Date:**												

**Warm-up:**						

RESISTANCE EXERCISE: Target = 4 sets x 10 repetition maximum

	Reps	Wt	Reps	Wt	Reps	Wt	Reps	Wt	Reps	Wt	Reps	Wt
Leg Press												
Chest Press												
Leg Curl												
Seated Row												
Leg Extension												
Biceps Curl												
Calf Raise												
Tricep Ext.												

AEROBIC EXERCISE: Target = 30 mins Constant Intensity

	Time	Level	Time	Level	Time	Level	Time	Level	Time	Level	Time	Level
**Treadmill**												
**Rower**												
**Cross-Trainer**												
**Bicycle**												

**Stretching:**						

**Session RPE**						
**# in group**						

Comments / Reasons for Missing Sessions:

NAME: ____________________________________ DOB: ____________________ **WEEKS 13 - 14**

**Session #:**	**37**		**38**		**39**		**40**		**41**		**42**	
**Day|Date:**												

**Warm-up:**						

RESISTANCE EXERCISE: Target = 4 sets x 8 repetition maximum *increase weights*

	Reps	Wt	Reps	Wt	Reps	Wt	Reps	Wt	Reps	Wt	Reps	Wt
Leg Press												
Chest Press												
Leg Curl												
Seated Row												
Leg Extension												
Biceps Curl												
Calf Raise												
Tricep Ext.												

AEROBIC EXERCISE: Target = 25 mins Constant Intensity

and 5 mins High Intensity Intervals (1-4 mins for each interval)

	Time	Level	Time	Level	Time	Level	Time	Level	Time	Level	Time	Level
**Treadmill**												
Intervals												
# of intervals												
**Rower**												
Intervals												
# of intervals												
**Cross-Trainer**												
Intervals												
# of intervals												
**Bicycle**												
Intervals												
# of intervals												

**Stretching:**						

**Session RPE**						
**# in group**						

Comments / Reasons for Missing Sessions:

NAME: ______________________________ DOB: __________________ **WEEKS 15 - 16**

**Session #:**	**43**		**44**		**45**		**46**		**47**		**48**	
**Day|Date:**												

**Warm-up:**						

RESISTANCE EXERCISE: Target = 4 sets x 8 repetition maximum

	Reps	Wt	Reps	Wt	Reps	Wt	Reps	Wt	Reps	Wt	Reps	Wt
Leg Press												
Chest Press												
Leg Curl												
Seated Row												
Leg Extension												
Biceps Curl												
Calf Raise												
Tricep Ext.												

AEROBIC EXERCISE: Target = 20-25 mins Constant Intensity

and 5-10 mins High Intensity Intervals (1-4 mins for each interval)

	Time	Level	Time	Level	Time	Level	Time	Level	Time	Level	Time	Level
**Treadmill**												
Intervals												
# of intervals												
**Rower**												
Intervals												
# of intervals												
**Cross-Trainer**												
Intervals												
# of intervals												
**Bicycle**												
Intervals												
# of intervals												

**Stretching:**						

**Session RPE**						
**# in group**						

Comments / Reasons for Missing Sessions:

NAME: ________________________ **DOB**: ______________ **WEEKS 17 - 18**

**Session #:**	**49**		**50**		**51**		**52**		**53**		**54**	
**Day|Date:**												

**Warm-up:**						

RESISTANCE EXERCISE: Target = 4 sets x 8 repetition maximum

	Reps	Wt	Reps	Wt	Reps	Wt	Reps	Wt	Reps	Wt	Reps	Wt
Leg Press												
Chest Press												
Leg Curl												
Seated Row												
Leg Extension												
Biceps Curl												
Calf Raise												
Tricep Ext.												

AEROBIC EXERCISE: Target = 20-25 mins Constant Intensity
and 5-10 mins High Intensity Intervals (1-4 mins for each interval)

	Time	Level	Time	Level	Time	Level	Time	Level	Time	Level	Time	Level
**Treadmill**												
Intervals												
# of intervals												
**Rower**												
Intervals												
# of intervals												
**Cross-Trainer**												
Intervals												
# of intervals												
**Bicycle**												
Intervals												
# of intervals												

**Stretching:**						

**Session RPE**						
**# in group**						

Comments / Reasons for Missing Sessions:

NAME: ___________________________________ **DOB:** ____________________ **WEEKS 19 - 20**

**Session #:**	**55**		**56**		**57**		**58**		**59**		**60**	
**Day|Date:**												

**Warm-up:**						

RESISTANCE EXERCISE: Target = 4 sets x 6 repetition maximum *increase weights*

	Reps	Wt	Reps	Wt	Reps	Wt	Reps	Wt	Reps	Wt	Reps	Wt
Leg Press												
Chest Press												
Leg Curl												
Seated Row												
Leg Extension												
Biceps Curl												
Calf Raise												
Tricep Ext.												

AEROBIC EXERCISE: Target = 20 mins Constant Intensity
and 10 mins High Intensity Intervals (1-4 mins for each interval)

	Time	Level	Time	Level	Time	Level	Time	Level	Time	Level	Time	Level
**Treadmill**												
Intervals												
# of intervals												
**Rower**												
Intervals												
# of intervals												
**Cross-Trainer**												
Intervals												
# of intervals												
**Bicycle**												
Intervals												
# of intervals												

**Stretching:**						

**Session RPE**						
**# in group**						

Comments / Reasons for Missing Sessions:

NAME: ________________________________ DOB: ____________________ **WEEKS 21 - 22**

**Session #:**	**61**		**62**		**63**		**64**		**65**		**66**	
**Day|Date:**												

**Warm-up:**						

RESISTANCE EXERCISE: Target = 4 sets x 6 repetition maximum

	Reps	Wt	Reps	Wt	Reps	Wt	Reps	Wt	Reps	Wt	Reps	Wt
Leg Press												
Chest Press												
Leg Curl												
Seated Row												
Leg Extension												
Biceps Curl												
Calf Raise												
Tricep Ext.												

AEROBIC EXERCISE: Target = 20 mins Constant Intensity
and 10 mins High Intensity Intervals (1-4 mins for each interval)

	Time	Level	Time	Level	Time	Level	Time	Level	Time	Level	Time	Level
**Treadmill**												
Intervals												
# of intervals												
**Rower**												
Intervals												
# of intervals												
**Cross-Trainer**												
Intervals												
# of intervals												
**Bicycle**												
Intervals												
# of intervals												

**Stretching:**						

**Session RPE**						
**# in group**						

Comments / Reasons for Missing Sessions:

NAME: ______________________________ **DOB**: __________________ **WEEKS 23 - 24**

**Session #:**	**67**		**68**		**69**		**70**		**71**		**72**	
**Day|Date:**												

**Warm-up:**						

RESISTANCE EXERCISE: Target = 4 sets x 6 repetition maximum

	Reps	Wt	Reps	Wt	Reps	Wt	Reps	Wt	Reps	Wt	Reps	Wt
Leg Press												
Chest Press												
Leg Curl												
Seated Row												
Leg Extension												
Biceps Curl												
Calf Raise												
Tricep Ext.												

AEROBIC EXERCISE: Target = 20 mins Constant Intensity
and 10 mins High Intensity Intervals (1-4 mins for each interval)

	Time	Level	Time	Level	Time	Level	Time	Level	Time	Level	Time	Level
**Treadmill**												
Intervals												
# of intervals												
**Rower**												
Intervals												
# of intervals												
**Cross-Trainer**												
Intervals												
# of intervals												
**Bicycle**												
Intervals												
# of intervals												

**Stretching:**						

**Session RPE**						
**# in group**						

Comments / Reasons for Missing Sessions:

SESSION RATING OF PERCEIVED EXERTION (RPE)

6	No exertion at all
7	Extremely light
8	
9	Very light
10	
11	Light
12	
13	Somewhat hard
14	
15	Hard (heavy)
16	
17	Very hard
18	
19	Extremely hard
20	Maximal exertion
