# Supplementary material for: Exercise and Psychosexual Education to Improve Sexual Function in Men With Prostate Cancer: A Randomized Clinical Trial
Source: JAMA Netw Open. 2025 Mar 12;8(3):e250413. doi: 10.1001/jamanetworkopen.2025.0413 (PMC11904736; doi:10.1001/jamanetworkopen.2025.0413)
Supplement: Supplement 2. — eTable 1. Summary of Adverse Events (Baseline to 6 Months) eTable 2. Subanalyses: Prostatectomy Cohort eTable 3. Subanalyses: Radiotherapy Cohort eTable 4. Subanalyses: ADT Cohort [file jamanetwopen-e250413-s002.pdf]

# Supplementary Online Content

Galvão DA, Newton RU, Taaffe DR, et al. Exercise and psychosexual education to improve sexual function in men with prostate cancer: a randomized clinical trial. *JAMA Netw Open.* 2025;8(3):e250413. doi:10.1001/jamanetworkopen.2025.0413

**eTable 1.** Summary of Adverse Events (Baseline to 6 Months)

**eTable 2.** Subanalyses: Prostatectomy Cohort

**eTable 3.** Subanalyses: Radiotherapy Cohort

**eTable 4.** Subanalyses: ADT Cohort

This supplementary material has been provided by the authors to give readers additional information about their work.

© 2025 Galvão DA et al. *JAMA Network Open*.

## eTable 1. Summary of Adverse Events (Baseline to 6 Months)

| | Exercise (n = 39) | Exercise + Psychosexual (n = 36) | Usual care (n = 37) |
|---|---|---|---|
| **Patients with at least one exercise-/treatment-emergent adverse event, n (%)** | 12 (31) [a] | 3 (8) [b] | 6 (16) [c] |
| **Musculoskeletal and connective tissue disorders, n (%)** | | | |
| Shoulder pain | 4 (10) | 1 (3) | 0 |
| Knee pain | 4 (10) | 1 (3) | 0 |
| Muscle spasm/muscle pain | 3 (8) | 1 (3) | 0 |
| Muscle strain | 1 (3) | 1 (3) | 0 |
| Lower back pain | 1 (3) | 0 | 0 |
| Ankle pain | 0 | 1 (3) | 0 |
| Hip pain | 1 (3) | 0 | 0 |
| Elbow pain | 1 (3) | 0 | 0 |
| Bone pain | 0 | 0 | 1 (3) |
| Joint inflammation | 1 (3) | 0 | 0 |
| **General disorders, n (%)** | | | |
| Fatigue | 0 | 0 | 3 (8) |
| **Injury, n (%)** | | | |
| Fall | 0 | 0 | 2 (5) |
| **Respiratory, thoracic, and mediastinal disorders, n (%)** | | | |
| Dyspnoea | 0 | 0 | 1 (3) |
| **Nervous system disorders, n (%)** | | | |
| Headache | 0 | 0 | 1 (3) |
| **Vascular disorders, n (%)** | | | |
| Hypertension | 0 | 0 | 1 (3) |
| **Infections, n (%)** | | | |
| Pneumonia | 0 | 0 | 1 (3) |

As per the National Cancer Institute's Common Terminology Criteria for Adverse Events (CTCAE, V.5.0), all recorded adverse events were categorised as non-serious (Grade 1-2) except for a fall in the usual care group that was considered serious (Grade 3).

[a] Two patients and one patient in the Exercise group reported 2 and 3 different adverse events, respectively.

[b] Two patients in the Exercise + Psychosexual group reported 2 different adverse events.

[c] One patient in the Usual Care group reported 5 different adverse events.

© 2025 Galvão DA et al. *JAMA Network Open*.

## eTable 2. Subanalyses: Prostatectomy Cohort

| Outcome | Baseline | Post-Test | Adjusted group difference | *P* value [a] |
|---|---|---|---|---|
| | Mean (SD) | Mean (SD) | Mean (95% CI) | |
| **IIEF Erectile function** | | | | |
| *Exercise and Exercise + Psychosexual (n=42)* | 4.6 (4.0) | 9.6 (8.6) | 1.6 (-2.5 to 5.7) | 0.36 |
| *Usual care (n=21)* | 5.4 (5.1) | 8.6 (9.6) | | |
| **IIEF Orgasmic function** | | | | |
| *Exercise and Exercise + Psychosexual* | 2.5 (2.7) | 3.7 (3.3) | 0.7 (-0.7 to 2.1) | 0.27 |
| *Usual care* | 2.9 (2.9) | 3.3 (3.6) | | |
| **IIEF Sexual desire** | | | | |
| *Exercise and Exercise + Psychosexual* | 4.9 (2.4) | 5.3 (2.4) | 0.2 (-0.7 to 1.1) | 0.69 |
| *Usual care* | 4.5 (2.2) | 4.9 (2.4) | | |
| **IIEF Intercourse satisfaction** | | | | |
| *Exercise and Exercise + Psychosexual* | 1.8 (3.2) | 4.2 (4.6) | 1.5 (-0.6 to 3.6) | 0.21 |
| *Usual care* | 2.9 (4.0) | 3.3 (4.2) | | |
| **IIEF Overall satisfaction** | | | | |
| *Exercise and Exercise + Psychosexual* | 3.6 (1.9) | 5.2 (2.0) | -0.0 (-1.1 to 1.1) | 0.87 |
| *Usual care* | 4.6 (2.1) | 5.5 (2.3) | | |
| **EPIC Sexual function** | | | | |
| *Exercise and Exercise + Psychosexual* | 18.6 (16.0) | 27.5 (22.7) | 6.2 (-3.4 to 15.8) | 0.28 |
| *Usual care* | 17.5 (16.5) | 19.8 (21.0) | | |
| **PR25 Sexual activity** | | | | |
| *Exercise and Exercise + Psychosexual* | 39.3 (24.4) | 41.7 (23.9) | 4.3 (-5.4 to 13.9) | 0.91 |
| *Usual care* | 40.5 (23.9) | 37.9 (20.8) | | |

Sub-analyses - Prostatectomy cohort may have also had radiation/ADT.
ANCOVA adjusted for age, sexual activity, previous radiation, previous/current ADT, and baseline value of the outcome.
[a] Statistical analysis based on log(x) or log(x+2) transformed data.

© 2025 Galvão DA et al. *JAMA Network Open*.

## eTable 3. Subanalyses: Radiotherapy Cohort

| Outcome | Baseline | Post-Test | Adjusted group difference | *P* value [a] |
|---|---|---|---|---|
| | Mean (SD) | Mean (SD) | Mean (95% CI) | |
| **IIEF Erectile function** | | | | |
| *Exercise and Exercise + Psychosexual (n=43)* | 5.3 (5.6) | 9.8 (9.2) | 4.2 (0.4 to 8.0) | 0.11 |
| *Usual care (n=19)* | 6.6 (7.7) | 6.3 (7.7) | | |
| **IIEF Orgasmic function** | | | | |
| *Exercise and Exercise + Psychosexual* | 2.5 (3.1) | 3.6 (3.7) | 0.7 (-0.8 to 2.3) | 0.54 |
| *Usual care* | 1.9 (2.8) | 2.3 (3.0) | | |
| **IIEF Sexual desire** | | | | |
| *Exercise and Exercise + Psychosexual* | 4.0 (2.1) | 4.8 (2.3) | 0.3 (-0.8 to 1.3) | 0.64 |
| *Usual care* | 3.9 (1.6) | 4.3 (1.8) | | |
| **IIEF Intercourse satisfaction** | | | | |
| *Exercise and Exercise + Psychosexual* | 2.2 (3.5) | 4.1 (4.6) | 1.6 (-0.4 to 3.5) | 0.13 |
| *Usual care* | 1.8 (3.1) | 2.0 (3.5) | | |
| **IIEF Overall satisfaction** | | | | |
| *Exercise and Exercise + Psychosexual* | 3.7 (2.0) | 5.2 (2.3) | 0.7 (-0.5 to 2.0) | 0.23 |
| *Usual care* | 4.2 (1.8) | 4.6 (2.3) | | |
| **EPIC Sexual function** | | | | |
| *Exercise and Exercise + Psychosexual* | 16.8 (16.4) | 25.6 (22.9) | 7.3 (-3.6 to 18.3) | 0.36 |
| *Usual care* | 18.0 (18.2) | 18.6 (22.0) | | |
| **PR25 Sexual activity** | | | | |
| *Exercise and Exercise + Psychosexual* | 31.8 (21.5) | 34.1 (23.5) | -2.7 (-13.0 to 7.6) | 0.66 |
| *Usual care* | 22.8 (15.9) | 30.0 (25.2) | | |

Sub-analyses - Radiotherapy cohort = Previous/current radiation incl. brachytherapy; may have also had prostatectomy/ADT. ANCOVA adjusted for age, sexual activity, previous prostatectomy, previous/current ADT, and baseline value of the outcome.
[a] Statistical analysis based on log(x) or log(x+2) transformed data.

© 2025 Galvão DA et al. *JAMA Network Open*.

## eTable 4. Subanalyses: ADT Cohort

| Outcome | Baseline | Post-Test | Adjusted group difference | *P* value [a] |
|---|---|---|---|---|
| | Mean (SD) | Mean (SD) | Mean (95% CI) | |
| **IIEF Erectile function** | | | | |
| *Exercise and Exercise + Psychosexual (n=43)* | 4.7 (4.6) | 8.8 (8.9) | 4.4 (0.2 to 8.7) | 0.08 |
| *Usual care (n=21)* | 5.9 (7.4) | 4.5 (6.0) | | |
| **IIEF Orgasmic function** | | | | |
| *Exercise and Exercise + Psychosexual* | 2.3 (2.9) | 3.2 (3.6) | 0.7 (-0.8 to 2.2) | 0.47 |
| *Usual care* | 1.1 (2.5) | 1.5 (2.4) | | |
| **IIEF Sexual desire** | | | | |
| *Exercise and Exercise + Psychosexual* | 3.8 (1.7) | 4.4 (2.2) | 0.5 (-0.5 to 1.6) | 0.40 |
| *Usual care* | 3.4 (1.6) | 3.5 (1.4) | | |
| **IIEF Intercourse satisfaction** | | | | |
| *Exercise and Exercise + Psychosexual* | 1.9 (2.8) | 3.7 (4.5) | 1.8 (-0.3 to 3.9) | 0.11 |
| *Usual care* | 1.2 (2.6) | 1.3 (2.7) | | |
| **IIEF Overall satisfaction** | | | | |
| *Exercise and Exercise + Psychosexual* | 3.7 (2.1) | 4.7 (2.4) | 0.9 (-0.4 to 2.2) | 0.21 |
| *Usual care* | 3.6 (1.7) | 3.8 (1.8) | | |
| **EPIC Sexual function** | | | | |
| *Exercise and Exercise + Psychosexual* | 13.8 (14.2) | 23.0 (22.5) | 8.7 (-2.6 to 20.0) | 0.29 |
| *Usual care* | 11.1 (14.3) | 11.0 (15.9) | | |
| **PR25 Sexual activity** | | | | |
| *Exercise and Exercise + Psychosexual* | 29.5 (22.1) | 30.8 (20.6) | 1.2 (-9.2 to 11.7) | 0.70 |
| *Usual care* | 16.7 (15.8) | 21.6 (22.9) | | |

Sub-analyses - ADT cohort = All participants with previous/current ADT; may have also had prostatectomy/radiation.
ANCOVA adjusted for age, sexual activity, previous prostatectomy, previous radiation, and baseline value of the outcome.
[a] Statistical analysis based on log(x) or log(x+2) transformed data.

© 2025 Galvão DA et al. *JAMA Network Open*.
