# Supplementary material for: Exercise and Psychosexual Education to Improve Sexual Function in Men With Prostate Cancer: A Randomized Clinical Trial
Source: JAMA Netw Open. 2025 Mar 12;8(3):e250413. doi: 10.1001/jamanetworkopen.2025.0413 (PMC11904736; doi:10.1001/jamanetworkopen.2025.0413)
Supplement: Supplement 3. — Data Sharing Statement [file jamanetwopen-e250413-s003.pdf]

Data Sharing Statement

Galvão. Exercise and Psychosexual Education on Sexual Function in Men With Prostate Cancer. *JAMA Netw Open*. Published March 12, 2025.
doi:10.1001/jamanetworkopen.2025.0413

Data
Additional Information: Improving sexual health in men with prostate cancer: randomised controlled trial of exercise and psychosexual therapies ACTRN12613001179729, https://www.anzctr.org.au https://www.anzctr.org.au/Trial/Registration/TrialReview.aspx?id=365201
Data available: Yes
Data types: Deidentified participant data
How to access data: Data available upon reasonable request d.galvao@ecu.edu.au
When available: With publication

Supporting Documents
Document types: None

Additional Information
Who can access the data: Anyone requesting the data subjective to proposal approval.
Types of analyses: Specific purposes subjective to approval.
Mechanisms of data availability: After approval of a proposal.
Any additional restrictions: N/A.
